# High-resolution summer precipitation variations in the western Chinese Loess Plateau during the last glacial

**DOI:** 10.1038/srep02785

**Published:** 2013-09-27

**Authors:** Zhiguo Rao, Fahu Chen, Hai Cheng, Weiguo Liu, Guo'an Wang, Zhongping Lai, Jan Bloemendal

**Affiliations:** 1 MOE Key Laboratory of Western China's Environmental Systems, Collaborative Innovation Centre for Arid Environments and Climate Change, Lanzhou University, Lanzhou 73000, China; 2 Institute of Global Environmental Change, Xi'an Jiaotong University, Xi'an 710054, China; 3 State Key Laboratory of Loess and Quaternary Geology, Institute of Earth Environment, Chinese Academy of Sciences, Xi'an 710075, China; 4 Department of Environmental Sciences and Technology, College of Resources and Environmental Sciences, China Agricultural University, Beijing 100193, China; 5 Cold and Arid Regions Environmental and Engineering Research Institute, Chinese Academy of Sciences, Lanzhou 730000, China; 6 Department of Geography, University of Liverpool, Liverpool L69 3BX, UK

## Abstract

We present a summer precipitation reconstruction for the last glacial (LG) on the western edge of the Chinese Loess Plateau (CLP) using a well-dated organic carbon isotopic dataset together with an independent modern process study results. Our results demonstrate that summer precipitation variations in the CLP during the LG were broadly correlated to the intensity of the Asian summer monsoon (ASM) as recorded by stalagmite oxygen isotopes from southern China. During the last deglaciation, the onset of the increase in temperatures at high latitudes in the Northern Hemisphere and decline in the intensity of the East Asia winter monsoon in mid latitudes was earlier than the increase in ASM intensity and our reconstructed summer precipitation in the western CLP. Quantitative reconstruction of a single paleoclimatic factor provides new insights and opportunities for further understanding of the paleoclimatic variations in monsoonal East Asia and their relation to the global climatic system.

Loess/paleosol sequences in the Chinese Loess Plateau (CLP), especially those with high accumulation rates in the northern and western CLP, are among the best terrestrial archives for late Quaternary paleoclimatic studies^1^,^2^. Paleoclimatic records in this area are extremely important for the understanding of the linkage and interplay between the evolution of the ASM at low latitudes^3^ and temperature variations at high latitudes^4^. However, until now, a high resolution paleoprecipitation reconstruction using a clear driving mechanism has been lacking in the western CLP.

Fractionation of carbon isotopes by modern C_3_ plants during CO_2_ uptake and fixation (Δ) can be described by mathematical models using the formula Δ = *a* + (*b* − *a*)(*p_i_*/*p_a_*), where *a* is the carbon isotopic fractionation during CO_2_ diffusion (ca. 4.4‰), *b* is the net fractionation caused by carboxylation (ca. 29‰) and *P*_i_ and *P*_a_ are the intercellular and ambient partial pressures of CO_2_, respectively^5^. In principle, increasing precipitation will result in more negative carbon isotopic composition (δ^13^C) values of C_3_ plants, due to the relatively high stomatal conductance present under a humid environment which will increase *P*_i_ and Δ^5^, and *vice versa*.

The relations between the δ^13^C of modern C_3_ plants and environmental factors have been widely studied and consistent results have been obtained which indicate that the δ^13^C of modern C_3_ plants responds principally to precipitation variations^6^,^7^,^8^ (Supplementary Figs. S1, S2 and S3). To a lesser extent, it also reflects more minor changes in temperature, altitude, and latitude^9^,^10^. Clearly, if the local terrestrial ecosystem was dominated by, or composed entirely of, C_3_ plants during a specific interval, the sedimentary δ^13^C data that derives from the local terrestrial biomass should be an indicator of precipitation. In Western Europe, total organic carbon isotopic data (δ^13^C_TOC_) of loess indicate a great predominance of C_3_ plants since the LG, and therefore these δ^13^C_TOC_ data have been used for precipitation reconstruction^11^,^12^.

During the past decade, the δ^13^C_TOC_ record of more than 10 Chinese loess/paleosol profiles has been studied (Fig. 1). The results demonstrate that, along the temporal sequence, the relative abundance of C_4_ plants increased from the LG to the Holocene (Supplementary Fig. S4); and that, along a spatial gradient, relative C_4_ plant abundance decreased gradually northwestwards (Supplementary Fig. S5)^13^,^14^,^15^,^16^,^17^, as is the case today^18^. During the LG, δ^13^C_TOC_ data from the high-temperature eastern CLP (to the east of the Liupan Mts. Fig. 1) indicate that this area was dominated by C_3_ plants with a minor increase in C_4_ plants during marine isotope stage 3^14^,^17^ (Supplementary Fig. S6), which means that the vegetation in the western CLP during the LG could be expected to be predominantly or entirely composed of C_3_ plants (Supplementary Fig. S6).

The Yuanbao profile (YB, 103.63°E, 35.15°N, 2,040 m a.s.l.), close to the Tibetan Plateau, is located on the western edge of the CLP (Fig. 1) with modern mean annual temperature (MAT) and precipitation (MAP) of ca. 6.8°C and ca. 500 mm, respectively. The Jingyuan profile (JY, 104.6°N, 36.35°N, 2,210 m a.s.l.), close to the Tengger Desert, is located on the northwestern edge of the CLP (Fig. 1) with modern MAP and MAT of ca. 238 mm and ca. 5.2°C, respectively. At both sites, precipitation primarily occurs during May to September (ca. 80% of MAP) with higher temperatures (also the main growing season of the local terrestrial vegetation), consistent with the modern climate in this area as controlled principally by the Asian monsoon (Supplementary Fig. S7 and S8). High resolution optically stimulated luminescence (OSL) dating clearly demonstrates that the loess/paleosol sequences of YB^19^,^20^ and JY^21^,^22^ have accumulated continuously since the LG without significant hiatuses. Previous studies have demonstrated that the loess/paleosol sequences located in the western CLP, including YB and JY, have great potential for high-resolution paleoclimatic reconstruction during the LG^21^,^22^,^23^. The top 25.74 m of YB was sampled for δ^13^C_TOC_ study at a 4 cm interval (ca. 100 years per sample)^24^; and the top ca. 30 m of JY was sampled at a 40 cm interval (ca. 1000 years per sample) for δ^13^C_TOC_ study^25^.

High resolution δ^13^C_TOC_ data (Fig. 2) from YB indicated that the study site was dominated by C_3_ plants during the LG and the relative abundance of C_4_ plants slightly increased during the Holocene period^24^,^26^ (Supplementary Fig. S6). δ^13^C_TOC_ data from JY (Fig. 2) indicated that the vegetation in the study site was dominated by C_3_ plants during both the LG and the Holocene^25^ (Supplementary Fig. S6). These results are consistent with the previous finding that the relative abundance of C_4_ plants decreased northwestwards across the entire CLP^13^,^14^,^15^,^16^,^17^,^18^ (Supplementary Fig. S4, S5). All of this evidence demonstrates that the δ^13^C_TOC_ data from YB during the LG and those from JY since the LG are suitable for paleoprecipitation reconstruction and that the results from both sites can be directly compared.

For paleoprecipitation reconstruction, the quantitative relations between δ^13^C of modern C_3_ plants and precipitation are established directly from modern case studies^6^,^7^,^8^,^9^. However, although almost all of the relevant studies have shown a negative correlation between the δ^13^C of C_3_ plants and precipitation, the magnitude of the correlation varies between different C_3_ plant species and in different regions^6^,^7^,^8^,^9^ (Supplementary Fig. S2, S3 and Table S1). Here we have used the results of δ^13^C_TOC_ measurements of 196 modern surface soils from arid central Asia^27^,^28^ as our point of reference (Supplementary Fig. S9). Our selection is based on: (i) the close location of these surface soils to the JY and YB sites; and (ii) the fact that both investigation on modern plants^29^ and the δ^13^C_TOC_ data of these surface soils^27^,^28^ (most of them < −24‰) demonstrate that the modern terrestrial ecosystem in this area is greatly dominated by C_3_ plants (Supplementary Fig. S9). Considering that the δ^13^C of modern C_3_ plants in arid central Asia principally responds to summer precipitation and the constrained uncertainties of the linear correlation between averaged surface soil δ^13^C_TOC_ values of 19 weather stations and the summer precipitation amount (May to Sept.) as recorded by the corresponding weather stations, we chose the correspondingly quantitative relation^27^ for the reconstruction of summer precipitation (Supplementary Fig. S9). Because the carbon isotopic composition of organic matter becomes more positive after long-term decomposition^30^, we have selected 1‰ as the magnitude of the baseline value for the δ^13^C_TOC_ data of loess/paleosol samples relative to their original values at the time of deposition. The secondary effect of temperature and atmospheric CO_2_ concentrations on C_3_ plant δ^13^C has been neglected. Based on these assumptions our final expression for summer precipitation reconstruction is: summer precipitation (mm) = −58*(*δ*^13^C_TOC_*1000 − 1) − 1266.5.

Correspondingly, the 95% confidence interval of the relevant linear correlation^27^ is used for the uncertainty estimation of the summer precipitation reconstruction (Supplementary Fig. S9).

## Results

In spite of the different resolutions, a high variability occurs in summer precipitation reconstructions of both the YB and JY sites, apparently consistent with the characteristics of modern climate in the study area (Supplementary Fig. S10); this indicates the high sensitivity of carbon isotopes of local terrestrial vegetation to variations in summer precipitation.

**Figure 1:**
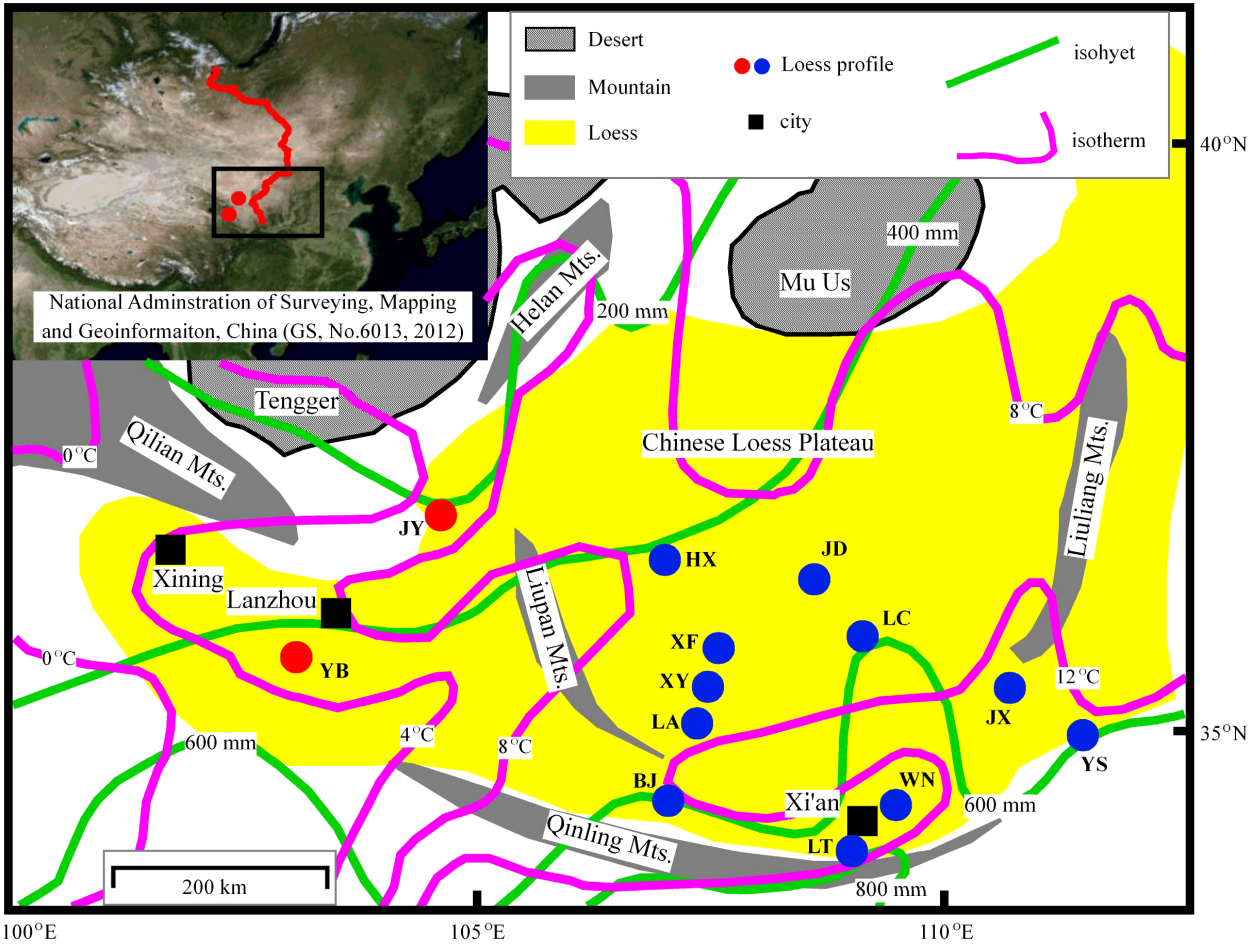
Locations of the Chinese loess/paleosol profiles that are cited in this paper and their basic climatic background^26^. Solid red line in the top left corner shows the distribution of the surface soils for δ^13^C_TOC_ study in arid central Asia^28^, adopted in this paper as modern quantitative relations reference^27^; the original image in the top left corner was downloaded from the public service website of the National Administration of Surveying, Mapping and Geoinformation, China (http://www.tianditu.cn/map/index.html). The solid red dots indicate the locations of the Yuanbao (YB) and Jingyuan (JY) profiles, and solid blue dots indicate the locations of the loess profiles in the eastern CLP for δ^13^C_TOC_ studies^13^,^14^,^15^,^16^,^17^. For the codes and δ^13^C_TOC_ records of these profiles, please refer to Supplementary Fig. S4, S5 and S6.

Our results indicate that summer precipitation varied from ca. 20 mm to 150 mm with uncertainties of ca. 70 ∼ 80 mm at the JY site during the past 70 ka with most values during the LG of less than 100 mm. Summer precipitation decreased gradually from MIS3 to an extremely arid MIS2, and then increased towards the Holocene (Supplementary Fig. S11). Considering the location of the JY profile so close to the Tengger Desert (Fig. 1), an extremely arid climate at the JY site during MIS2 is reasonable. Although there are only a few data values for the Holocene, the results indicate that the maximum summer precipitation during the Holocene was more than 150 mm (with uncertainty of ca. 70 mm), which is close to the modern summer precipitation in the JY area (ca. 190 mm, averaged from 1961 to 1990) (Supplementary Fig. S10).

Reconstructed summer precipitation at the YB site during the LG varied mainly from ca. 150 mm to 350 mm with uncertainties of ca. 70 mm (Supplementary Fig. S11, Table S2). Modern summer precipitation in the YB area is ca. 400 mm (averaged from 1961 to 1990, Supplementary Table S3), greater than that of the JY site. During the LG, reconstructed summer precipitation at YB was generally higher than that at JY (Supplementary Fig. S10), suggesting that the modern climatic gradient between the JY and YB sites is consistent with that during the LG, which supports our methodology.

**Figure 2:**
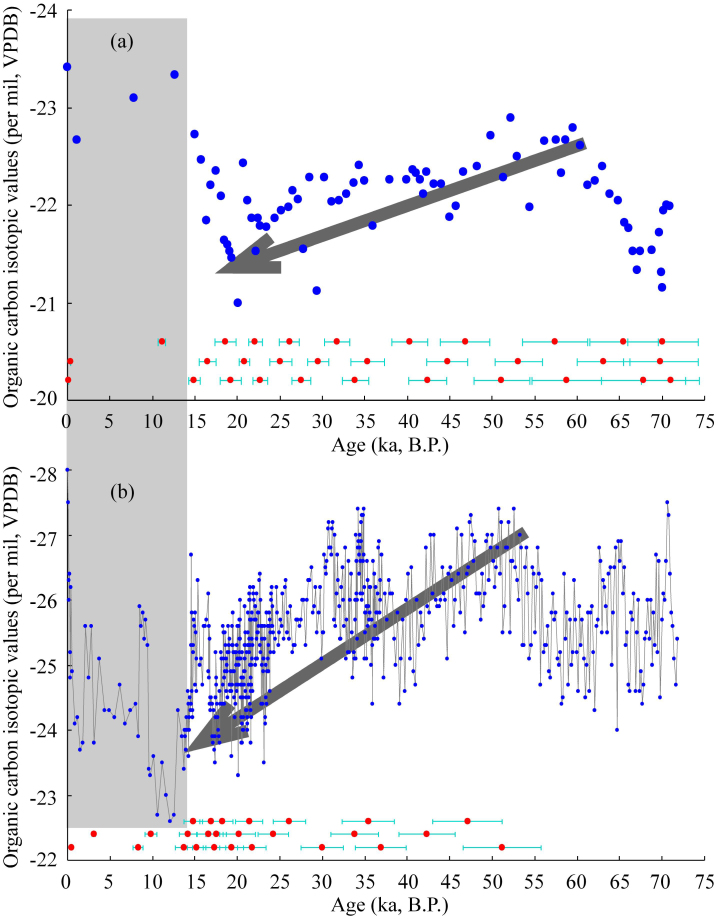
Loess δ^13^C_TOC_ data of the JY (a) and YB (b) profiles plotted against ages. Red dots represent the OSL data of the JY^21^,^22^ and YB^19^,^20^ profiles, with errors represented by light blue bars. The age series of both profiles were obtained by linear interpolation of these OSL data. Bold grey arrows indicate the same trend in loess δ^13^C_TOC_ data of the JY and YB profiles during the LG. The most important and opposite trend occurs during the transition from the LG to the Holocene, which indicates a relative increase in abundance of C_4_ plants towards the Holocene at the YB site (also in Supplementary Fig. S6).

Loess δ^13^C_TOC_ data of the YB profile during the Holocene are apparently contaminated by C_4_ plants, especially during the early Holocene (Fig. 2, Supplementary Fig. S6), and this is the essential reason why a reconstruction of summer precipitation for the whole Holocene at the YB site has not been completed. However, if loess δ^13^C_TOC_ data of the topmost 2 samples (−28‰ and −27.5‰ respectively, Supplementary Table S2) of the YB profile were to be used for the calculation of summer precipitation, then summer precipitation levels of ca. 415 mm and 385 mm (with uncertainties of ca. 70 mm) respectively would be obtained, consistent with modern summer precipitation at the YB site of an averaged value of ca. 400 mm; these findings also support our methodology.

A series of rapid climatic events during the LG has been recorded at the YB site using a grain size proxy for winter monsoon intensity^23^. These events may be associated with temperature variations at high latitudes of the Northern Hemisphere *via* a relation between the intensity of the winter monsoon and the north westerlies^1^,^21^,^22^,^23^. Based on the corresponding North Greenland Ice Core Project (NGRIP) ice-core ages (GICC05^31^) of Heinrich and interstadial events, we adjusted the ages of the YB winter monsoon record based on their correlation with NGRIP^4^ oxygen isotopic (δ^18^O) record (Supplementary Fig. S12). There is an overall consistency between the OSL dating results of the YB profile and the transferred NGRIP ice-core ages, with relatively younger (ca. 4 ∼ 5 ka) OSL ages^19^,^20^ occurring between 20 ka to 60 ka (Supplementary Fig. S13).

**Figure 3:**
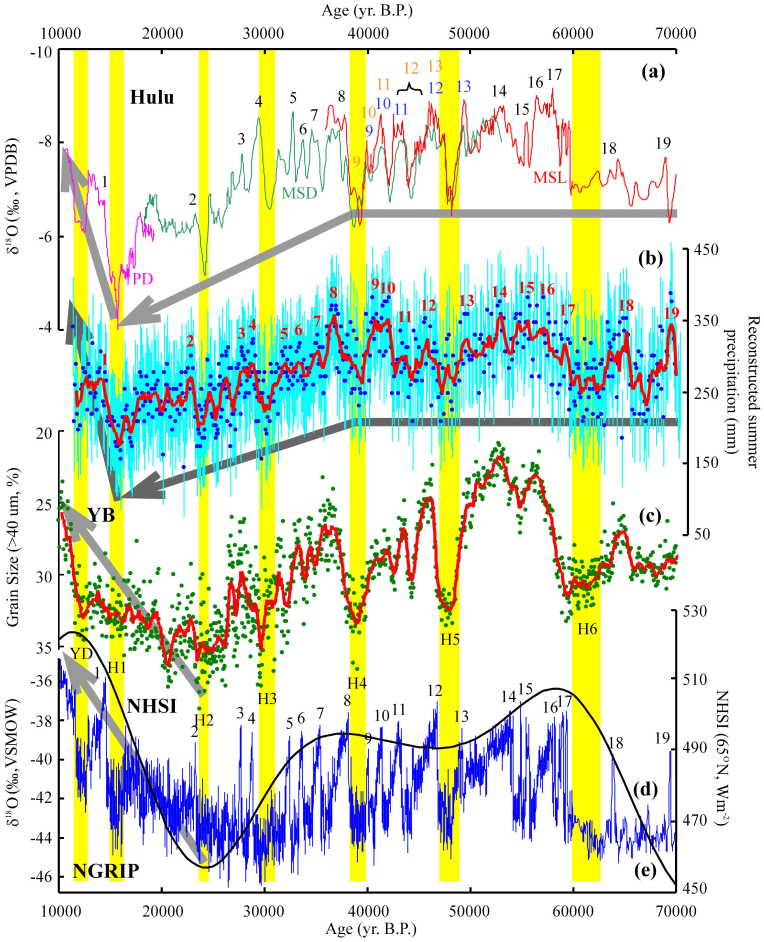
Comparison of the summer precipitation reconstruction results of the YB profile and other records. (a) Hulu stalagmite δ^18^O record^3^ (purple-PD, green-MSD, red-MSL) from southern China; (b) reconstructed summer precipitation at the YB site during the LG (blue dots represent the calculated values of summer precipitation, the light blue bars represent the uncertainties of the corresponding reconstructed summer precipitation, the solid red line represents the 500-years-time-window running averaged values, and the original data is shown in Supplementary Table S2); (c) grain size data of the YB profile (green dots represent the original values, the solid red line represents the 500-years-time-window running averaged values, and the original data is shown in Supplementary Table S2); (d) the Northern Hemisphere solar insolation^34^ (NHSI, black line, 65°N, average value of June); (e) NGRIP ice-core δ^18^O record^4^ (blue line, with GICC05 chronology^31^). Black Arabic numerals indicate the interstadial events, and the yellow vertical bars indicate the YD and Heinrich events. Gray arrows demonstrate the overall trends. Age series of the YB records were transferred from the NGRIP ice core (Supplementary Fig. S12).

Summer precipitation along the adjusted NGRIP ice-core age series at the YB site shows a high degree of consistency with the stalagmite δ^18^O record from Hulu Cave^3^ in southern China. Both data sets record Heinrich events 1 to 6 and interstadial events 1 to 19 (Fig. 3); in general, arid events, represented by sharply decreased summer precipitation in the western CLP, broadly correspond to a weakened ASM recorded in stalagmite δ^18^O data from southeastern China^3^, and to cold events in high northern latitudes recorded in the NGRIP δ^18^O data^4^. More importantly, from the H4 to H1 events, the stalagmite δ^18^O data gradually became more positive and the summer precipitation in the YB area also decreased gradually (Fig. 3). Assuming that the stalagmite δ^18^O data from southern China have recorded variations in the intensity of the ASM^3^, and based on the comparison of the YB and Hulu records, it appears that the decrease in the intensity of the ASM was the direct cause of the decrease in summer precipitation in northwest China.

## Discussion

The assumption that the Chinese stalagmite δ^18^O data record the intensity of the ASM has been widely debated^32^,^33^. For example, in a recent comparative simulation study of the Last Glacial Maximum (LGM) and Heinrich event 1 (H1), changes in the Indian monsoon that are controlled by the sea surface temperature (SST) of the Indian Ocean have been suggested as the main cause of the stalagmite δ^18^O variations in China^33^. Due to the lack of direct evidence that the Indian Ocean SST during H1 was lower than during the LGM, and based on the high degree of consistency between the variations in our reconstructed summer precipitation and those in the southern Chinese stalagmite δ^18^O data (Fig. 3), our results demonstrate that, at least during the LG period, variations in the Chinese stalagmite δ^18^O data records are a valid indicator of the intensity of the ASM.

During the transition from the LG to the Holocene, summer precipitation in the YB area and the intensity of the ASM^3^ decreased gradually. Subsequently, an enhancement of both the ASM and summer precipitation occurred around the time of the H1 event (Fig. 3). The onset of the rise of temperature at high latitudes recorded by the NGRIP δ^18^O data^4^ occurred at around the time of the H2 event or earlier (Fig. 3). Grain size data from the YB profile indicate that the onset of the decrease of the East Asian winter monsoon was also coeval with the H2 event^23^ (Fig. 3), which is also consistent with grain size records from the Luochuan^1^ and JY^22^ profiles. During the transition from the LG to the Holocene, it seems that both high latitude temperatures^4^ and the mid latitude East Asian winter monsoon^1^,^22^,^23^ responded rapidly to increasing insolation^34^ (Fig. 3). However, the response of the low latitude ASM was apparently delayed^3^. All of this evidence indicates more complex driving mechanisms for the low latitude summer monsoon than for the mid and high latitude climatic components.

In conclusion, our results are significant in the following respects: 1) because previous studies have already demonstrated that the local biomass on the western edge of the CLP^24^,^25^,^26^ and in the loess area in Western Europe^11^,^12^ was dominated by C_3_ plants since the LG, sedimentary δ^13^C_TOC_ derived therefrom may be a valid indicator of paleoprecipitation; and therefore our work provides a new paradigm for paleoprecipitation reconstruction in this vast region between Western Europe and the western CLP and the regions to their north; 2) the paleoclimatic significance of the Chinese stalagmite δ^18^O data has been widely debated^32^,^33^; however, we demonstrated that at least during the LG, the Chinese stalagmite δ^18^O data are a credible indicator of the intensity of the ASM. Further work needs to be done to test the validity of this finding over longer timescales; and 3) the combination of future independent paleotemperature reconstructions and our paleoprecipitation reconstruction results may constitute a major advance in paleoclimatic studies of monsoonal East Asia.

## Methods

The YB profile was sampled at 2 cm intervals (ca. 50 years per sample) for the top 25.74 m. After removal of organic matter and carbonate with HCl (∼10%) and H_2_O_2_ (∼10%), the grain size of all the samples was measured using a Malvern Master Sizer 2000 laser diffraction analyser. The measurement range of this equipment is 0.02–2000 μm. In this paper we use the percentage of the grain size fraction > 40 μm as an indicator of the east Asian winter monsoon intensity and the strength of the north westerlies^1^,^21^,^22^,^23^. A 4 cm interval was used for the δ^13^C_TOC_ measurements. The samples were pretreated as follows: ∼ 10% hydrochloric acid (HCl) was used to remove carbonates, followed by washing with distilled water until the suspension was neutral. The wet samples were then sieved at 120 μm to remove tiny sand particles and gravels. After sieving, the samples were dried at 70°C. The gas collection method involved static combustion. The evolved CO_2_ was analyzed for δ^13^C using a Thermo Finnigan Delta Plus mass spectrometer. The standard materials used for the measurements were international standard tree-rings (Corundum balls, IAEA-C5). The mean value of 29 repeat measurements was −25.7‰ with a standard deviation of ± 0.13‰ (the reported value of the standard is −25.49 ± 0.72‰). Repeated measurements on both the standard materials and samples (81 times in total) showed that the experimental error is less than ± 0.2‰. (See references 19 and 20 for the OSL dating methods used on profile YB, and references 21 and 22 for those used on profile JY. See reference 25 for the organic carbon isotopic data for profile JY.)

## Author Contributions

F.H.C. designed the study and led the writing of the paper. Z.G.R. performed the organic carbon isotopic analysis of the YB profile, and contributed to data analysis, interpretation and paper writing. W.G.L. performed the organic carbon isotopic analysis of the JY profile. Z.P.L. conducted the OSL dating of the YB profile. All authors contributed to the discussion and interpretation of the results and to the writing of the manuscript.

## Supplementary Material

Supplementary Information Supplementary Information
