# Supplementary material for: High-resolution summer precipitation variations in the western Chinese Loess Plateau during the last glacial
Source: Sci Rep. 2013 Sep 27;3:2785. doi: 10.1038/srep02785 (PMC3784955; doi:10.1038/srep02785)
Supplement: Supplementary Information [file srep02785-s1.pdf]

Supplementary Information

High-resolution summer precipitation variations in the western Chinese Loess Plateau during the last glacial

Zhiguo Rao[1], Fahu Chen[1*], Hai Cheng[2], Weiguo Liu[3], Guo'an Wang[4], Zhongping Lai[5], Jan Bloemendal[6]

[1] MOE Key Laboratory of Western China's Environmental Systems, Collaborative Innovation Centre for Arid Environments and Climate Change, Lanzhou University, Lanzhou 73000, China;

[2] Institute of Global Environmental Change, Xi'an Jiaotong University, Xi'an 710054, China;

[3] State Key Laboratory of Loess and Quaternary Geology, Institute of Earth Environment, Chinese Academy of Sciences, Xi'an 710075, China;

[4] Department of Environmental Sciences and Technology, College of Resources and Environmental Sciences, China Agricultural University, Beijing 100193, China;

[5] Cold and Arid Regions Environmental and Engineering Research Institute, Chinese Academy of Sciences, Lanzhou 730000, China;

[6] Departement of Geography, University of Liverpool, Liverpool L69 3BX, UK

*Corresponding author: fhchen@lzu.edu.cn

Table of contents

Part 1, carbon isotopes of modern C_3 plants and precipitation

Part 2, C_3/C_4 variations in the CLP since the last glacial

Part 3, modern summer monsoon limit in East Asia

Part 4, surface soil $\delta^{13}C$ results from arid central Asia

Part 5, reconstruction of summer precipitation at the YB and JY sites

Part 6, refined age-model of the YB profile

Part 7, original data of the YB profile

Part 8, climate data from Jingyuan and Linxia (1961~1990)

Part 9, supplementary references

(Totally, 15 figures and 3 tables)

Part 1, carbon isotopes of modern C_3 plants and precipitation

Previous studies indicate that the $\delta^{13}C$ of modern C_3 plants responds mainly to the variation in local precipitation (Stewart et al., 1995). Recently, $\delta^{13}C$ data (n = 3310) for 334 woody plant species (all woody plants are C_3 species) at 105 sites on a global scale were systematically summarised, and a strong negative correlation was revealed between the $\delta^{13}C$ data and corresponding mean annual precipitation (MAP, Diefendorf et al., 2010). Similar results reported recently come from another review work on $\delta^{13}C$ data of modern C_3 plants (including trees, shrubs, herbs, and grasses) from ca. 570 individual sites, also at a global scale (Kohn, 2010).

In north China, $\delta^{13}C$ of 367 modern C_3 plant samples (mostly grasses) distributed in 15 sites in Chinese loess area have been measured (Wang et al., 2003). The averaged values of the 15 sites showed a significant negative correlation with corresponding MAP (a in Fig. S1). In the central CLP, along a south to north transect, foliar $\delta^{13}C$ of 121 C_3 plant samples from 41 dominant species (including trees, shrubs and grasses) in 18 families in 7 sites was studied (Zheng and Shangguan, 2007). Averaged $\delta^{13}C$ values of the 7 sites showed a significant negative correlation with local MAP also (b in Fig. S1).

All the above mentioned results indicate clearly a negative correlation between $\delta^{13}C$ of modern C_3 plants and precipitation. However, the obtained correlation coefficients are apparently different (as shown in Fig. S1). That may have resulted from the different sensitivity of $\delta^{13}C$ of different C_3 species to variations in precipitation. Detailed study results of the $\delta^{13}C$ in 3 modern C_3 species (*Stipa*

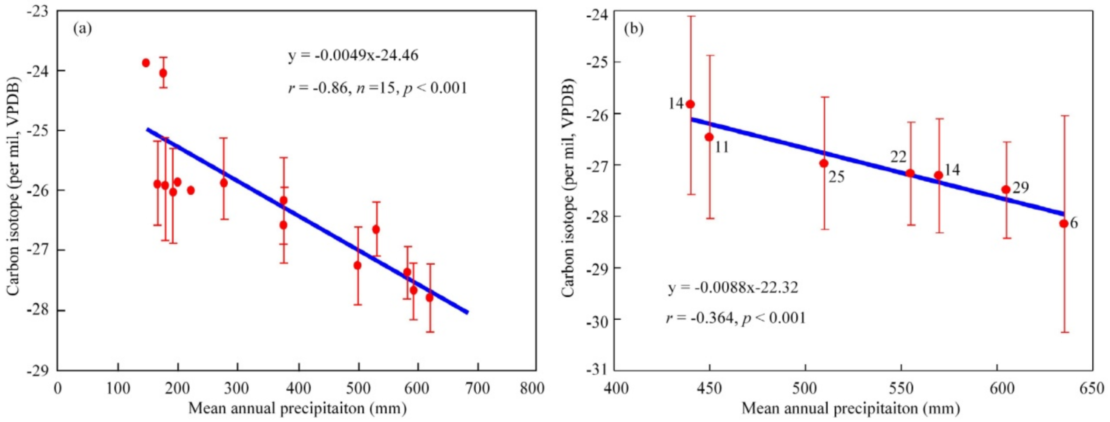

Figure S1 The negative correlation between MAP and the $\delta^{13}C$ of modern C_3 plants. (a) averaged values for 15 sites in the loess region of north China (Wang et al., 2003), the total number of data being 367; (b) averaged values of 7 sites from the central CLP (Zheng and Shangguan, 2007), the total number of data being 121; the number of data for each site is shown by Arabic numerals. Please note the different correlation coefficients.

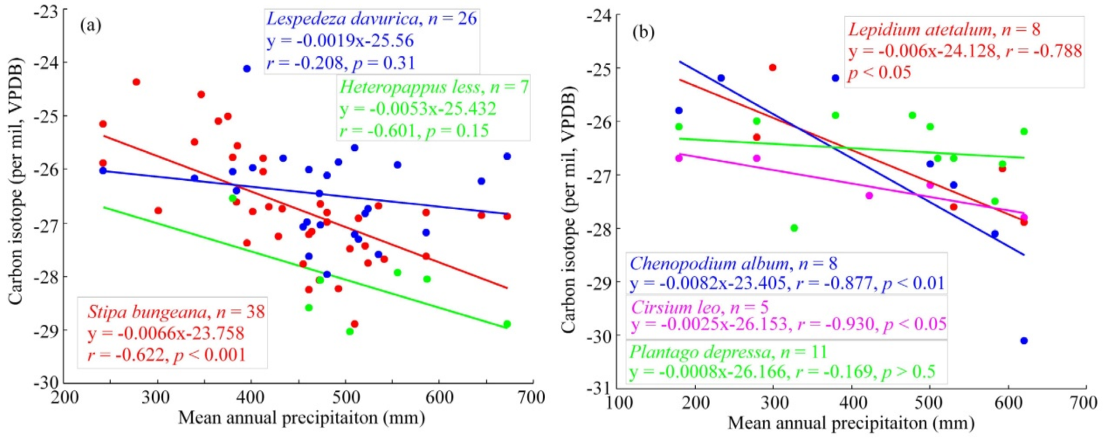

Figure S2 The detailed results of the negative correlation between MAP and the $\delta^{13}C$ of different modern C_3 species. (a) 3 modern C_3 species from northwest China (Liu et al., 2005a); (b) 4 modern C_3 species from northwest China (Wang and Han, 2001a). Please note the differences in the linear relations for different species indicated by different colours.

bungeana, *Lespedeza sp.* and *Heteropappus less*) in northwest China displays a completely different linear negative correlation with local MAP, the only significant correlation being with *Stipa bungeana* (Liu et al., 2005a; a in Fig. S2). Similar results

come from 4 modern C_3 species (*Plantago depressa*, *Lepidium apetalum*, *Chenopodium album* and *Cirsium leo*) also in northwest China (Wang and Han, 2001a; b in Fig. S2), only the correlation with *Plantago depressa* being insignificant. In a specific location near Baiyin City (also close to the Jingyuan loess profile mentioned in this paper) in northwest China, 7 modern C_3 species were sampled during late June and middle July, 1999. The corresponding weather data is shown in Table S1, and the $\delta^{13}C$ results are shown in Fig. S3. The results clearly indicate the negative responses of $\delta^{13}C$ in modern C_3 plants to increasing precipitation and the different sensitivities of the $\delta^{13}C$ in different C_3 species to identical variation in precipitation (Wang and Han, 2001b).

Table S1 Summer weather data for Baiyin city in 1999 (cited from Wang and Han, 2001b; please note the much higher rainfall in July, and the similar temperature and solar radiation values in June, July and August).

	**June**	**July**	**August**
**Rainfall (mm)**	32.6	**154**	13.8
**Temperature (°C)**	20.2	21.2	21.7
**Solar radiation (h)**	234.6	255	251

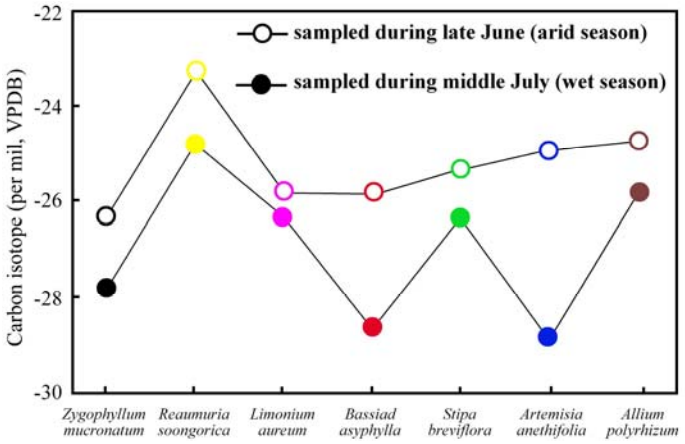

Figure S3 Comparative results of the $\delta^{13}C$ of 7 modern C_3 species sampled in late June and middle July in 1999 near Baiyin City (modified from Wang and Han, 2001b).

Obviously, the above results demonstrate the negative correlation between the $\delta^{13}C$ of modern C_3 plants and precipitation. However, due to the variable sensitivities of different C_3 species and the exact combination of C_3 species during geological periods, which is very difficult to determine, the results of the $\delta^{13}C$ in modern C_3 plants and precipitation cannot be used directly as a modern reference for paleoprecipitation reconstruction.

Part 2, C_3/C_4 variations in the CLP since the last glacial

During past decades, compound-specific $\delta^{13}C$ studies of long chain *n*-alkanes derived from terrestrial higher plants or/and $\delta^{13}C$ studies of total organic matter in more than 10 loess profiles (e.g., Gu et al., 2003; Zhang et al., 2003; Vidic and Montañez, 2004; Rao et al., 2005; Liu et al., 2005b, 2005c, 2011; Chen et al., 2006) have clarified the C_3/C_4 variations in the CLP since the last glacial , with most of the profiles located in the eastern CLP (east of the Liupan Mountains, Fig. 1) with only a few located in the western CLP (west of the Liupan Mountains, Fig. 1).

Generally speaking, the results demonstrate that, in profiles in the eastern CLP, the loess $\delta^{13}C_{TOC}$ data were more positive in paleosol layers formed during warmer and wetter Holocene climatic conditions and were more negative in loess layers accumulated under arid and colder last glacial climate conditions (Rao et al., 2006), indicating an increase of C_4 relative abundance from the last glacial to the Holocene as climatic conditions became warmer and wetter (Fig. S4).

Correspondingly, with the climate gradually becoming arid and colder in the

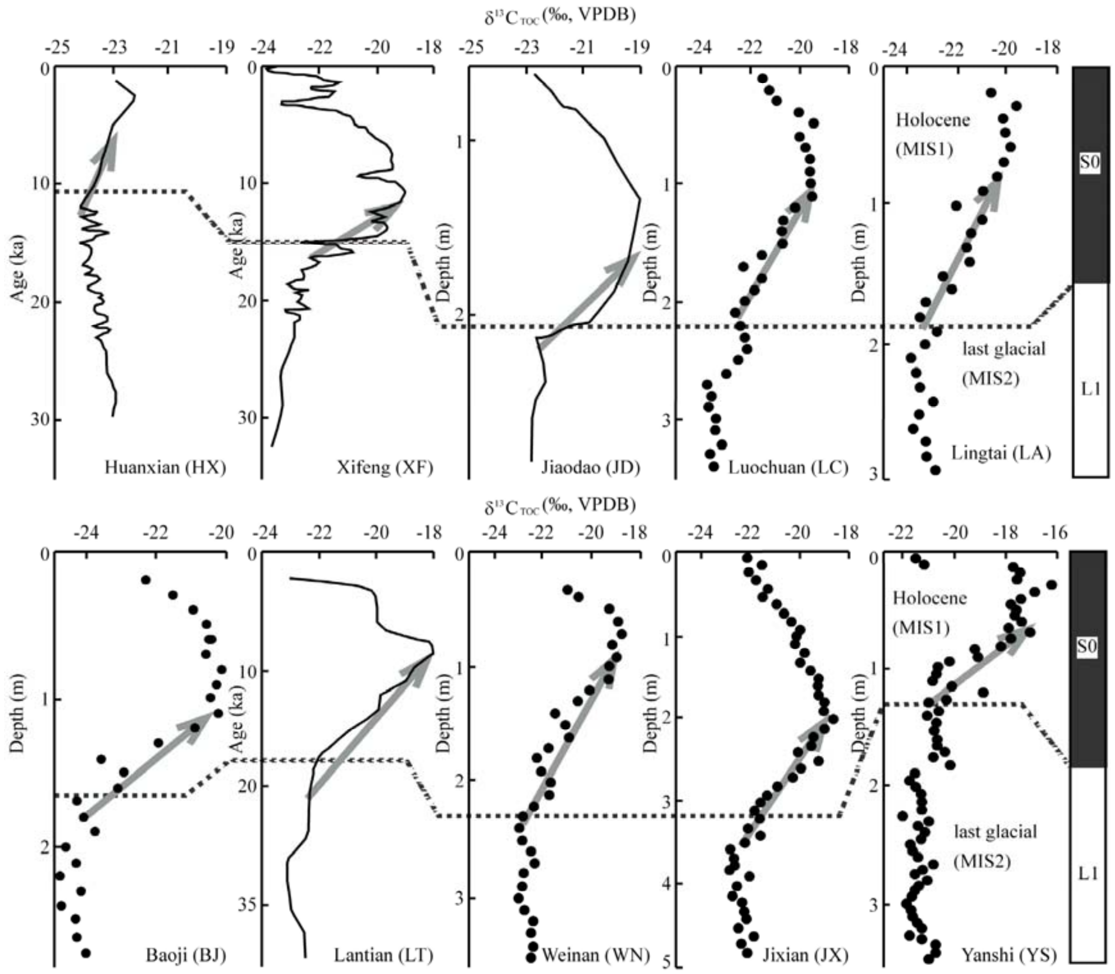

Figure S4 Variations in $\delta^{13}C_{TOC}$ in LC, LA, BJ, WN, JX, YS (Gu et al., 2003), HX, XF, LT (Liu et al., 2005b), and JD loess profile (Vidic and Montañez, 2004) since the last glacial. All the results indicate an increase in C_4 relative abundance since the last glacial to the Holocene, with more positive $\delta^{13}C_{TOC}$ values occurring in Holocene paleosol (S0) layers. (The locations of these profiles are shown in Fig. 1, and the codes for the loess profiles are identical as in Fig. 1).

Northwest CLP, a decrease in the relative abundance of C_4 plants would be expected (Rao et al., 2006). Comparison of the loess $\delta^{13}C_{TOC}$ data in the 3 profiles (Weinan, Lingtai and Huanxian located in the CLP along a transect from southeast to northwest, respectively), clearly demonstrates the decrease of C_4 relative abundance northwestward during both the last glacial and the Holocene, with the $\delta^{13}C_{TOC}$ data of both the last glacial loess layer and the Holocene paleosol layer becoming

increasingly negative towards the northwest (Fig. S5). Consistent with the decreasing trend of C_4 relative abundance in the CLP towards the northwest during both the last glacial and the Holocene, the loess $\delta^{13}C_{TOC}$ data from the Yuanbao (YB) profile located in the westernmost CLP indicates that the local terrestrial vegetation during the last glacial was dominated by C_3 plants with only a negligible C_4 contribution (Rao et al., 2005; Chen et al., 2006). Similarly, the loess $\delta^{13}C_{TOC}$ data from the Jingyuan (JY) profile that located in the northwesternmost CLP indicates that the local terrestrial vegetation since the last glacial was dominated by C_3 plants with only a negligible C_4 contribution (Liu et al., 2011, Fig. S6).

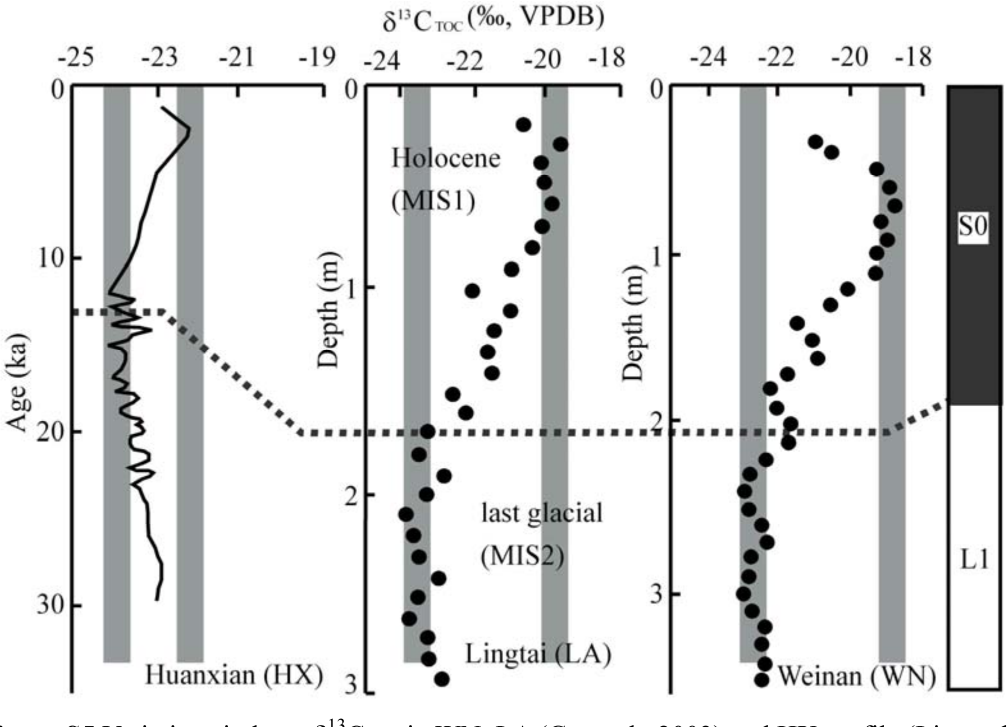

Figure S5 Variations in loess $\delta^{13}C_{TOC}$ in WN, LA (Gu et al., 2003) and HX profile (Liu et al., 2005b) along a spatial gradient. The results indicate that the relative abundance of C_4 plants decreased from southeast to northwest in the CLP during both the Holocene and the last glacial, with $\delta^{13}C_{TOC}$ data in both Holocene paleosol (S0) and last glacial loess (L1) layers decreasing gradually towards the northwest.

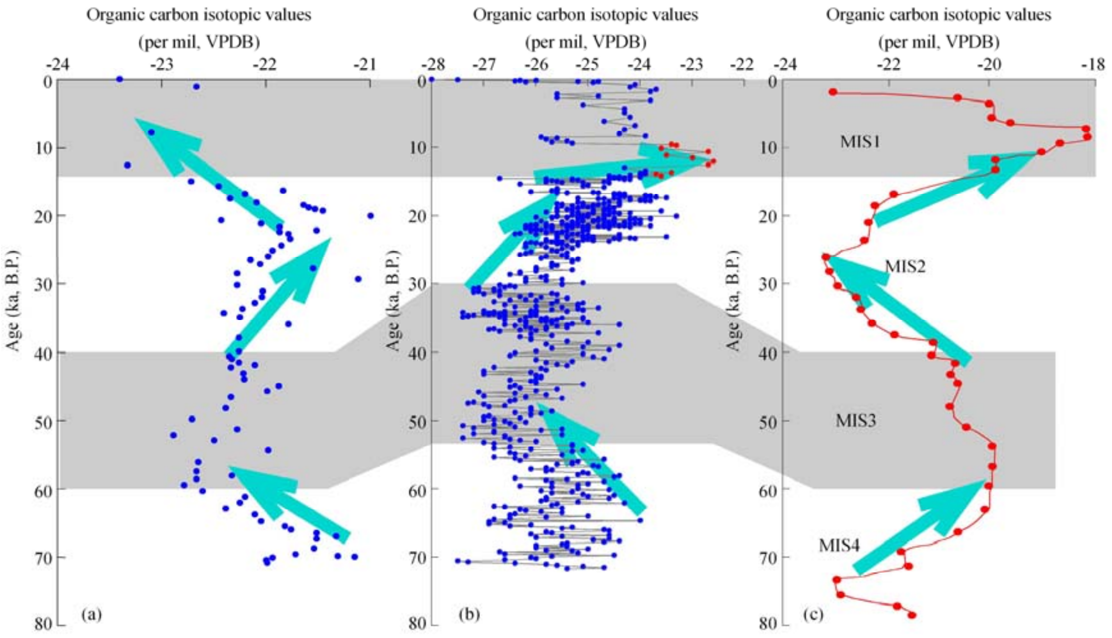

Figure S6 Comparison of $\delta^{13}C_{TOC}$ records from the JY profile (a) located in the northwesternmost CLP (Liu et al., 2011),the YB profiles (b) located in the westernmost CLP (this study), and the LT profile (c) located in the southernmost CLP (Liu et al., 2005b), See Figure 1 for site locations. The age series of the JY profile is a linear interpolation of OSL data from Sun et al., 2010, 2012; the YB profile series is a linear interpolation of OSL data from Lai and Wintle, 2006 and Lai et al., 2007; and the LT profile series is taken directly from Liu et al., 2005b. On a glacial/interglacial timescale, the $\delta^{13}C_{TOC}$ data for the Holocene paleosol at the JY site were more negative than for the last glacial loess, which is converse to the results from the other profiles (see Figures S4 and S5). This indicates that the $\delta^{13}C_{TOC}$ data for the JY do not record variations in C_3/C_4 relative abundance but, rather, record $\delta^{13}C$ variations of C_3 plants since the last glacial. During the last glacial, $\delta^{13}C_{TOC}$ data for JY and YB were more negative in the weakly developed paleosol layer formed during MIS3 than in the loess layers accumulated during MIS2 and MIS4, which is the converse of the results from the LT profile. This indicates that the terrestrial vegetation at the JY and YB sites during the last glacial was dominated by, or composed entirely of, C_3 plants. The overall trend of the loess $\delta^{13}C_{TOC}$ data is shown in bold light blue arrows for clear comparison. The loess $\delta^{13}C_{TOC}$ data from the YB profile during the early Holocene are emphasized by the comparative use of red dots indicating the LT profile data and the apparent C_4 contribution.

Clearly, the results shown above demonstrate that local terrestrial vegetation

during the last glacial at the YB site and since the last glacial at the JY site were dominated by C_3 plants with only a negligible C_4 contribution. In other words, loess $\delta^{13}C_{TOC}$ data of the last glacial at the YB site and since the last glacial at the JY site can be used for paleoprecipitation reconstruction by way of the modern relation between the $\delta^{13}C$ data of C_3 plants and precipitation.

Part 3, modern summer monsoon limit in East Asia

As shown in Figure S7, the YB and JY profiles are located in the frontier area of the modern Asian summer monsoon.

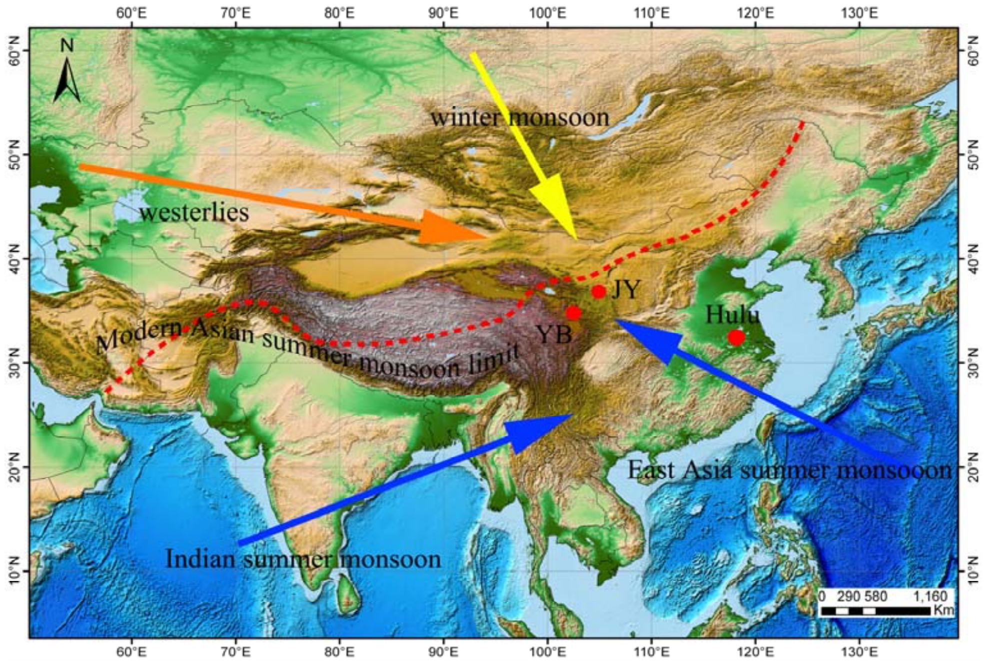

Figure S7 Locations of the YB and JY loess/paleosol profiles and the Hulu cave in southern China. The yellow arrow shows the approximate winter monsoon path; the brown arrow shows the westerlies and the blue arrow shows the East Asian and Indian summer monsoon paths. The dashed red line indicates the modern Asian summer monsoon limit (Chen et al., 2008). Clearly, the YB and JY profiles are located in the frontier region of the modern Asian summer monsoon. The original map was generated by ESRI ArcGIS (v9.1); for the source of the original data for this map please refer to Amante and Eakins, 2009.

Considering that the intensity of the summer monsoon gradually deceased during the late Holocene, as shown by a recently reported stalagmite oxygen isotopic record from Sanbao Cave (Fig. S8, Dong et al., 2010), it seems that the intensity of the most recent summer monsoon is very close to that during the Younger Dryas event (ca. 12 ka B.P.). However, there remains a significant distance between the modern summer monsoon limit and the location of the JY and YB sites, especially the latter (more than 250km), so it seems likely that the summer monsoon was also the major source of summer precipitation in the JY and YB sites during the last glacial.

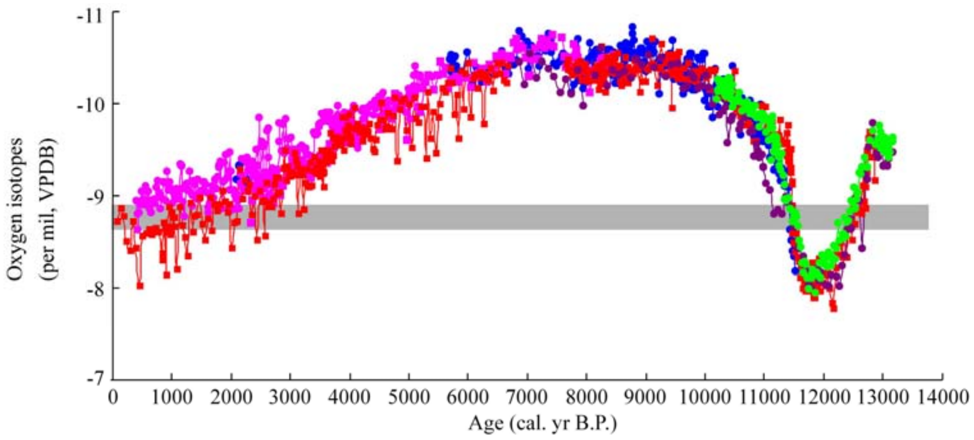

Figure S8 Stalagmite oxygen isotopic record for the last ca. 14, 000 years from the Sanbao Cave located in central China (Dong et al., 2010). Different colors represent data from different stalagmite samples. Note the horizontal grey bar, which provides a comparison of the most recent data with that of the YD event.

Part 4, surface soil $\delta^{13}C$ results from arid central Asia

As above mentioned in Supplementary Part 1, the relation between the $\delta^{13}C$ values of modern C_3 plants and precipitation cannot be used directly for paleoprecipitation reconstruction. Therefore, relations between surface soil $\delta^{13}C$

values and precipitation from an area with a full vegetation cover dominated by C_3 plants is a better choice as a modern reference for paleoprecipitation reconstruction. Given that such surface soil $\delta^{13}C$ values can represent the carbon isotopic signal of the overlying vegetation at an ecosystem level, the influence of different sensitivities of different C_3 plants to variations in precipitation can be largely avoided.

In arid central Asia, the $\delta^{13}C$ in 196 surface soil samples along a south to north transect were measured and reported (Lee et al., 2005; Feng et al., 2008; Fig. S9). Owing to the proximity of our profiles (YB and JY) to the surface soil transect (Fig. 1) and the considerable spatial gradient of the surface soil transect (very representative), we chose the relation between these surface soil $\delta^{13}C$ values and precipitation as a modern reference for paleoprecipitation reconstruction.

It has been widely recognized that temperature is the most important climatic factor controlling the growth of C_4 plants (Long, 1983; Rao et al., 2012). An investigation of modern plants on Gengga Mountain in southwestern China indicated that almost no C_4 plants have been observed above an altitude of ca. 2100m with a MAT of 9.4°C and a summer temperature of 15.3°C (Li et al., 2009). A similar investigation on Lingshan Mountain near Beijing city in north China demonstrated that almost no C_4 plants have been observed above an altitude of ca. 1800m (Wang et al., 2010). According to the results of Long, C_4 plants are extremely rare in areas with summer temperatures lower than 16°C (Long, 1983). Comparative analyses of surface soil $\delta^{13}C$ values at a continental scale from eastern China to Australia and the Great Plains of North America, with a MAT of ca. 12°C, has been found to be the "threshold

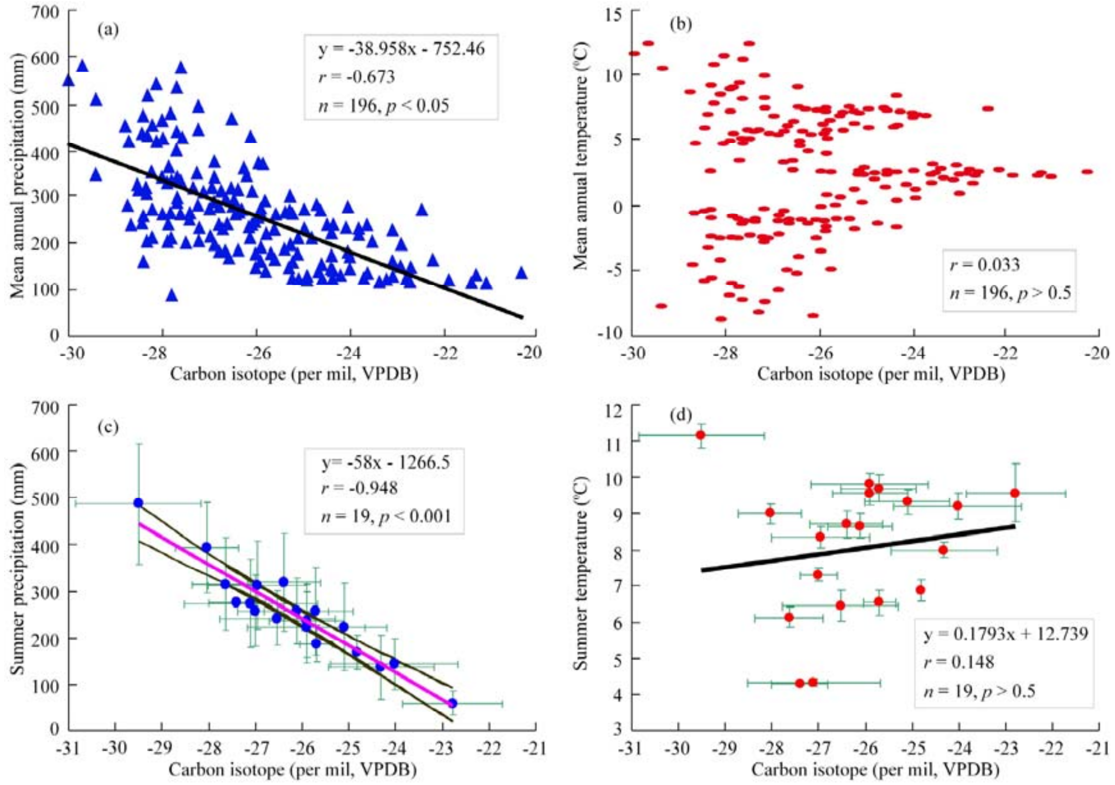

Figure S9 Relations between surface soil $\delta^{13}C_{TOC}$ data from arid central Asia and precipitation and temperature. (a) $\delta^{13}C_{TOC}$ in 196 modern surface soils plotted against MAP (Feng et al., 2008); (b) $\delta^{13}C_{TOC}$ in 196 modern surface soils plotted against MAT (Feng et al., 2008). A line is not shown because a linear relation is not apparent; (c) averaged $\delta^{13}C_{TOC}$ values of 19 sites (solid blue dots) plotted against summer precipitation (Lee et al., 2005); the linear negative correlation is represented by the purple line, and the black lines represent the 95% confidence interval of the linear correlation; (d) averaged $\delta^{13}C_{TOC}$ values of 19 sites plotted against summer temperature (Lee et al., 2005). Horizontal and vertical light blue bars in (c) and (d) represent the 1 σ standard deviations of the averaged surface soil $\delta^{13}C_{TOC}$ values and corresponding averaged climatic data (summer precipitation and temperature) respectively. See Fig. 1 for distribution of the surface soils. The quantitative relation between summer precipitation and averaged surface soil $\delta^{13}C_{TOC}$ data in (c) has been used as the modern reference for summer precipitation reconstruction in this work, with the assumption that the loess $\delta^{13}C_{TOC}$ data were systematically 1‰ more positive after long-term decomposition, and the 95% confidence interval of the linear correlation in (c) has been used to estimate the uncertainties of the summer precipitation reconstruction.

temperature" for the growth of C_4 plants (Rao et al., 2010). However, the MAT for the

196 surface soil samples from arid central Asia is almost entirely lower than 12°C (b in Fig. S9); therefore, the presence of significant C_4 plants in the study region in arid central Asia is out of the question. At the same time, surface soil $\delta^{13}C$ values for higher MAT of 10°C to 12°C in arid central Asia mainly range from -27‰ to -30‰ (b in Fig. S9), apparently falling into the $\delta^{13}C$ range of C_3 plants. Conversely, the positive surface soil $\delta^{13}C$ values of -20‰ to -24‰ may partially include carbon isotopic signals from C_4 plants; the corresponding MAT mainly ranges from 0°C to 5°C (b in Fig. S9), temperatures too low for the growth of C_4 plants. Our investigation on modern plants in arid central Asia indicates that there are only a few C_4 species distributed close to the rivers or with very short life-spans, such as chenopods (Dicots). Although we cannot completely preclude a contribution from C_4 plants in the $\delta^{13}C$ dataset from arid central Asia, and considering the significant influence of temperature on the growth of C_4 plants and the distribution of the $\delta^{13}C$ dataset along MAT in arid central Asia (b in Fig. S9), we firmly believe that the relative abundance of C_4 plants in the local biomass, or the contribution of C_4 plants to the surface soil $\delta^{13}C$ data, are extremely limited, and therefore negligible.

It should also be noted that our survey in the Linxia Basin in which the YB site is located (MAT of 6.8°C) demonstrates that there are only few C_4 species mainly around croplands and residential areas. Therefore, considering the lower temperature during the last glacial, it is reasonable to conclude that the contribution by C_4 plants to the YB loess $\delta^{13}C$ during the last glacial was negligible.

There seems to be an exponential relation between surface soil $\delta^{13}C$ values and

MAT in arid central Asia (b in Fig. S9). However, the positive surface soil $\delta^{13}C$ values around MAT of ca. 2.5°C mainly range from -20‰ to -24‰. The corresponding MAP for these positive $\delta^{13}C$ values (-20‰ to -24‰) mainly range from 100mm to 300mm (a in Fig. S9), falling into the climatic range of desert and Gobi in arid central Asia, and located in the middle of the studied transect (Fig. 1, Lee et al., 2005; Feng et al., 2008). Therefore, the exponential relation may just reflect the significant effect of precipitation on the plant $\delta^{13}C$ values from another aspect, rather than the significant effect of temperature. More importantly, the influence of temperature on plant $\delta^{13}C$ values is very complicated. Generally speaking, temperature can affect plant $\delta^{13}C$ *via* the effect of the stomatal conductance of the leaves and the bioactivity of the photosynthetic enzymes. Temperatures that are either too low or too high will restrain the stomatal conductance of the leaves and the bioactivity of the photosynthetic enzymes. Normally, the "transform temperature" lies between 20°C and 30°C or higher. Apparently, even considering that summer temperature is higher than MAT, the observed MAT of ca. 2.5°C (the corresponding summer temperature is 8°C to 10°C, Lee et al., 2005) is too low to be treated as the "transform temperature". Therefore, the exponential relation between MAT and the surface soil $\delta^{13}C$ values from arid central Asia (b in Fig. S9) is just presentational, not logical.

Although there is a significant negative correlation, there are huge uncertainties in the relation between the 196 surface soil $\delta^{13}C$ values from arid central Asia and MAP (Feng et al., 2008; a in Fig. S9). The uncertainties may mainly come from the following two considerations. 1) Differences in $\delta^{13}C$ values of surface soil underlying

different vegetation types and located in different topographical locations (such as sunny and shady slopes, hilltops and river valleys) in a small area with the approximately the same MAP. 2) The $\delta^{13}C$ values of terrestrial plants are mainly affected by precipitation during the growing seasons, i.e. summer precipitation in arid central Asia, not the annual precipitation (Lee et al., 2005). Therefore, the relation between averaged surface soil $\delta^{13}C$ values close to the 19 weather stations and corresponding summer (May to September) precipitation recorded by the weather stations is much more stable with constrained uncertainties (Lee et al., 2005; c in Fig. S9). In this paper, we select the quantitative relation between the averaged surface soil $\delta^{13}C$ values and summer precipitation (c in Fig. S9) as the modern reference for summer precipitation reconstruction with the assumption that loess $\delta^{13}C_{TOC}$ data were systematically 1‰ more positive after long-term decomposition. Based on the original data including the averaged $\delta^{13}C$ values and summer precipitation amount from the 19 weather stations, we calculated the 95% confidence interval (CI) of the linear relation (c in Fig. S9). Also, the quantitative estimation of the summer precipitation with a prediction interval (PI) at the 95% level was calculated using this relation and our $\delta^{13}C$ data during the last glacial derived from the YB site and since the last glacial derived from the JY site. The linear fitting, CI calculation, summer precipitation estimation, and PI calculation were all performed using the statistical package R, version 3.0.0 (R Core Team 2013).

Part 5, reconstruction of summer precipitation at the YB and JY sites

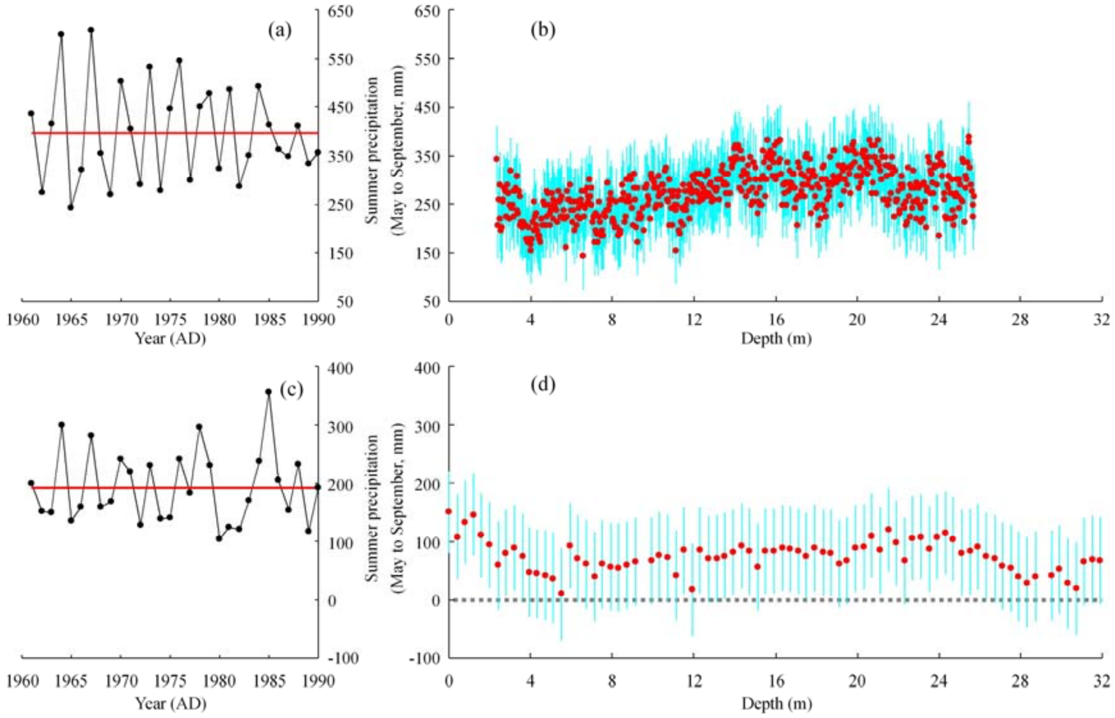

Figure S10 Comparison of modern summer precipitation (May to September) recorded at the Linxia station (a) and Jingyuan station (c) from 1961 to 1990 and the reconstructed summer precipitation of the YB (b) and JY (d) profiles plotted against depth. Horizontal red lines in (a) and (c) indicate the averaged values; solid red dots in (b) and (d) represent the calculated values of summer precipitation with the corresponding uncertainties represented by vertical light blue bars.

Comparison of the reconstructed summer precipitation of the YB and JY profiles (plotted against depth) and the modern summer precipitation (May to September from 1961 to 1990) at the Jingyuan and Linxia stations is shown in Fig. S10. Apparently, high variability exists in the modern summer precipitation at the Jingyuan (ca. 100 to ca. 350mm) and Linxia (ca. 240 to 610mm) sites (Fig. S10), consisting of common characteristics of the modern climate in the arid and semi-arid areas of central Asia. High variability also existed in the loess $\delta^{13}C_{TOC}$ data from the YB and JY profiles and the corresponding reconstructed summer precipitation, especially in the high-resolution data of the YB profile during the last glacial (Fig. S10), indicating the high

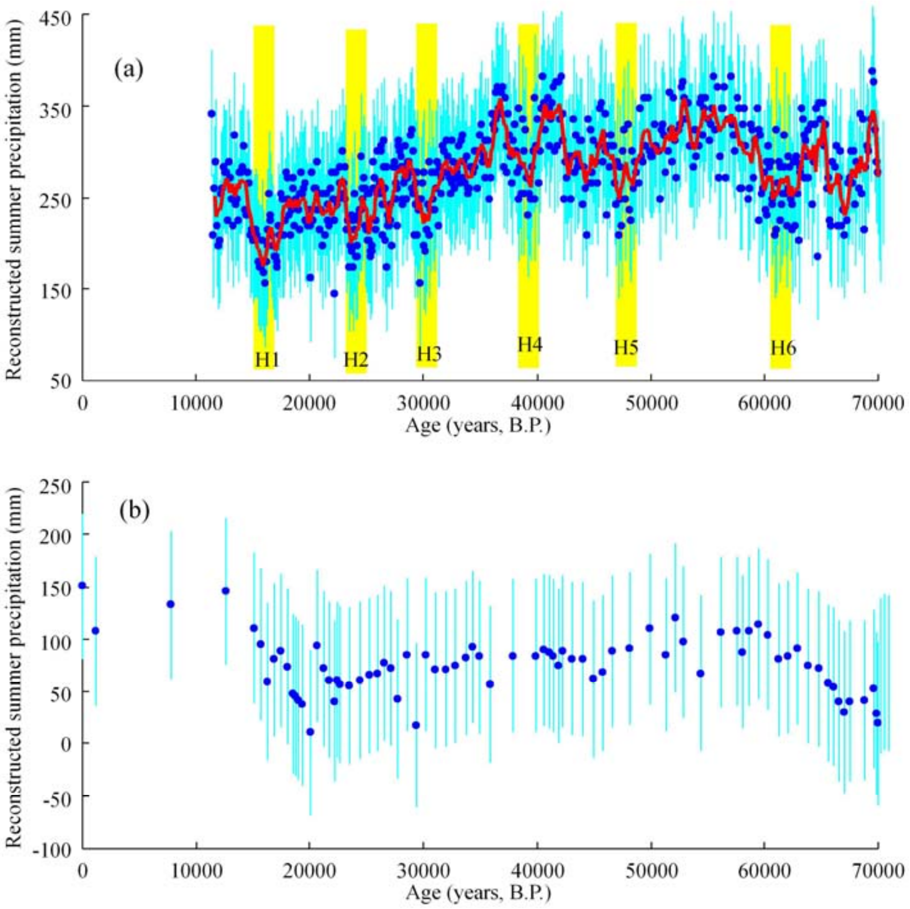

Figure S11 Comparison of reconstructed summer precipitation at the YB (a) and JY (b) sites *versus* age. Solid blue dots in both (a) and (b) represent the calculated values with uncertainties represented by light blue vertical bars. The solid red line in (a) represents the 500-year-runing averaged values and the vertical yellow bars in (a) represent the possible H events (H1 to H6). The age series in the YB profile is transferred from the NGRIP ice core based on the comparison of grain size data of the YB profile and oxygen isotopic data of NGRIP (NGRIP, 2004; please refer to Supplementary Part 6 for details). The age series in the JY profile is interpolated from its OSL dating results (Sun et al., 2010, 2012).

sensitivity to climatic changes of carbon isotopes of terrestrial vegetation in the arid and semi-arid areas (especially the variations in precipitation).

The most recent reconstructed summer precipitation for the JY site is ca. 150mm with an uncertainty of ca. 70mm, close to the modern averaged summer precipitation of the Jingyuan station of ca. 190mm (1961~1990). The Holocene loess $\delta^{13}C_{TOC}$ data

in the YB profile apparently contain a C_4 signal as shown in Fig. S6, especially during the early Holocene, that's why we abandoned the summer precipitation reconstruction of the entire Holocene at the YB site. The loess $\delta^{13}C_{TOC}$ data in the topmost 2 samples are -28‰ and -27.5‰, respectively (Table S2). If these two data are used to calculate the summer precipitation, results of ca. 415mm and 386mm, respectively, are obtained also with uncertainties of ca. 70mm , which is very close to the modern averaged summer precipitation at the Linxia station of ca. 400mm (1961~1990; Table S3). Comparison of the most recent calculated summer precipitation and the corresponding modern averaged value, the relatively greater difference in the JY profile apparently resulted from its relatively positive loess $\delta^{13}C_{TOC}$ data which fall into the most positive end of the surface soil $\delta^{13}C_{TOC}$ values in arid central Asia (Fig. S9), thus raising uncertainty. All this evidence validates our summer precipitation reconstruction method.

Detailed comparison of the reconstructed summer precipitation of the YB and JY profiles with the age sequences is impossible, due to the data resolution in the JY profile being too low. However, both datasets show higher summer precipitation from 30ka to 60ka (marine isotope stage 3, MIS3), followed by a decrease towards MIS2 (Fig. S11).

Part 6, refined age-model of the YB profile

The relatively huge errors of the OSL data from the YB profile, especially those during the last glacial (as shown in Fig. 2 in the main text) preclude the comparison of

high-resolution YB records with other records. For a long time (e.g. Porter and An, 1995), grain size data in the Chinese loess have been related to temperature variations at high latitudes in the northern hemisphere by way of variations in the intensity of the East Asian winter monsoon and the vigor of the westerly winds. During cold phases such as the Heinrich events, the enhanced winter monsoon and the northern hemisphere westerlies transported more coarse dust grains to the CLP, especially to its western sector because of its proximity to the deserts (Chen et al., 1997; Sun et al., 2010, 2012). This relation allows us to transfer the ice core ages from high latitudes in the northern hemisphere to the loess profile in the western CLP.

The grain size data in the YB profile (> 40μm, %, mainly reflecting the intensity of the winter monsoon) have been compared with the oxygen isotopic record of NGRIP (NGRIP, 2004; indicating the temperature variations in the high latitudes of the northern hemisphere) as a means of selecting appropriate age control points, mainly dependent on the cold events (Fig. S12). After that, the transferred age series in the YB profile was obtained by linear interpolation of the selected age control points. The results indicate that, between ca. 10 and 20ka, the OSL data were generally consistent with the transferred NGRIP ages; between ca. 20 and 60ka, it seems that the OSL data are systematically younger than the transferred NGRIP ages with an average offset of ca. 4~5ka (Fig. S13). This comparison further confirms the validity of transferring the NGRIP ice core ages to the YB loess profile based on comparison of the loess grain size and the NGRIP oxygen isotopic data (NGRIP,

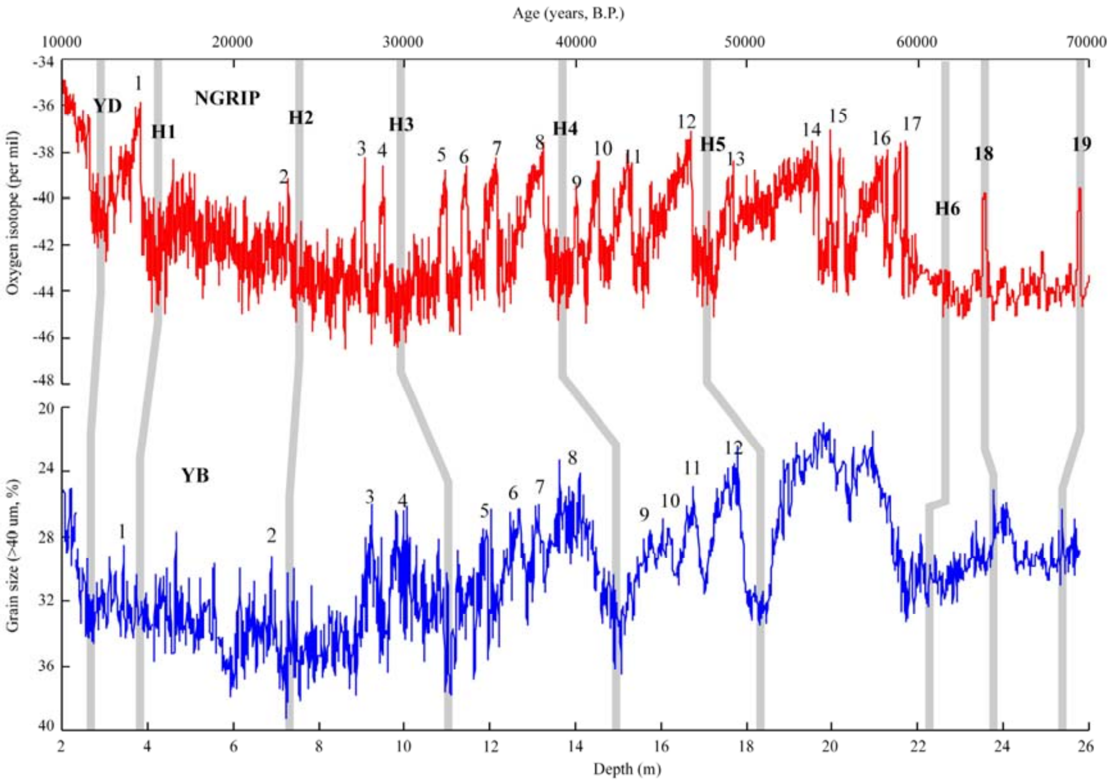

Figure S12 Comparison of high-resolution grain size data (blue lines) from the YB profile plotted against depth and oxygen isotopic data (red lines) from the NGRIP ice core (NGRIP, 2004) plotted against the age series of GICC05 (Svensson et al., 2008). Based on the comparison, 9 age control points (represented by the vertical grey bars) were selected in order to match the NGRIP age series to the YB profile by interpolation. Arabic numerals indicate interstadial events. YD is the Younger Dryas event and H1-H6 marks the Heinrich events. Please note the reversed grain size scale.

2004). Although the transferred age series is not accurate, and considering the widely accepted control mechanism of Chinese loess grain size, we chose the transferred age series from the NGRIP ice core as the final age model of the YB profile for comparing the YB records with others (Fig. 3 in main text).

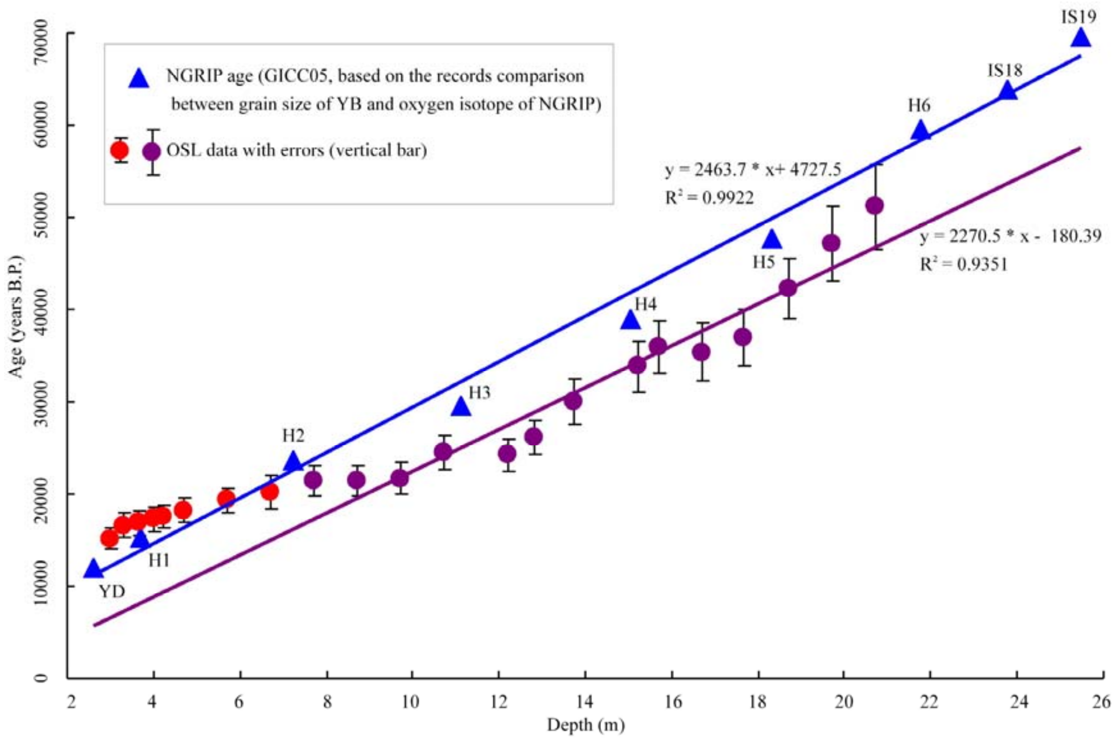

Figure S13 Comparison of the OSL data (solid red and purple dots with error bars, Lai and Wintle, 2006; Lai et al., 2007) and the selected 9 age control points (solid blue triangles) transferred from NGRIP plotted against depth of the YB profile. The purple line is based on part of the OSL data represented by solid purple dots to show the approximately systematic difference between OSL data and NGRIP data between 20ka and 60ka.

Part 7, original data of the YB profile

For an intuitive presentation, original data and reconstructed summer precipitation from the YB profile are plotted against depth and age series as shown in Figs.S14 and S15, respectively. Correspondingly, all relevant data from the YB profile are shown in Table S2.

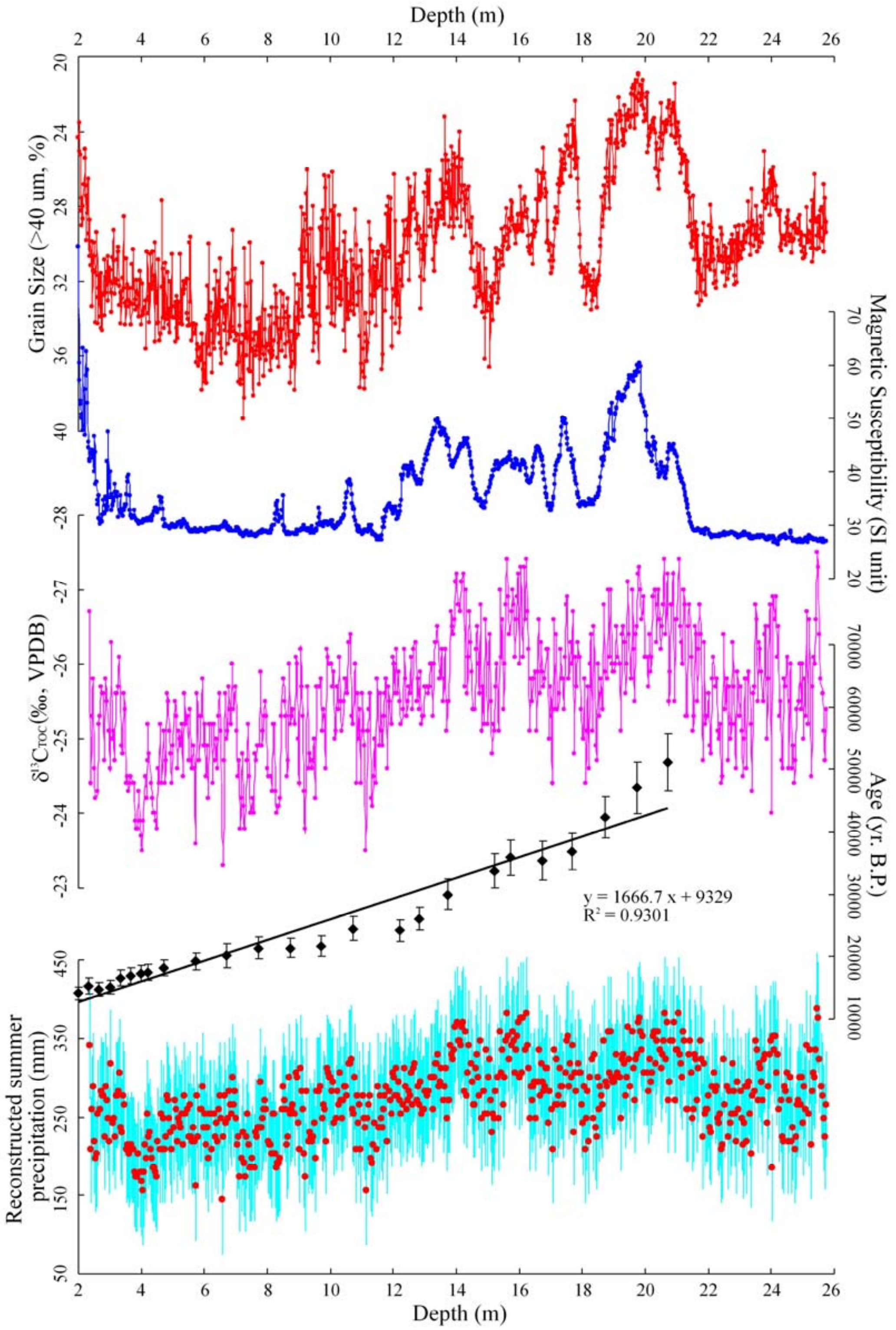

Figure S14 The original data from the YB profile plotted against depth. Red series are grain size data (>40μm, %); blue series are magnetic susceptibility (SI units); purple series are loess $\delta^{13}C_{TOC}$ data (‰, VPDB); black dots with error bars are the OSL dating results (same as in Fig. S13); calculated summer precipitation is shown by solid red dots with uncertainties represented by light blue vertical bars.

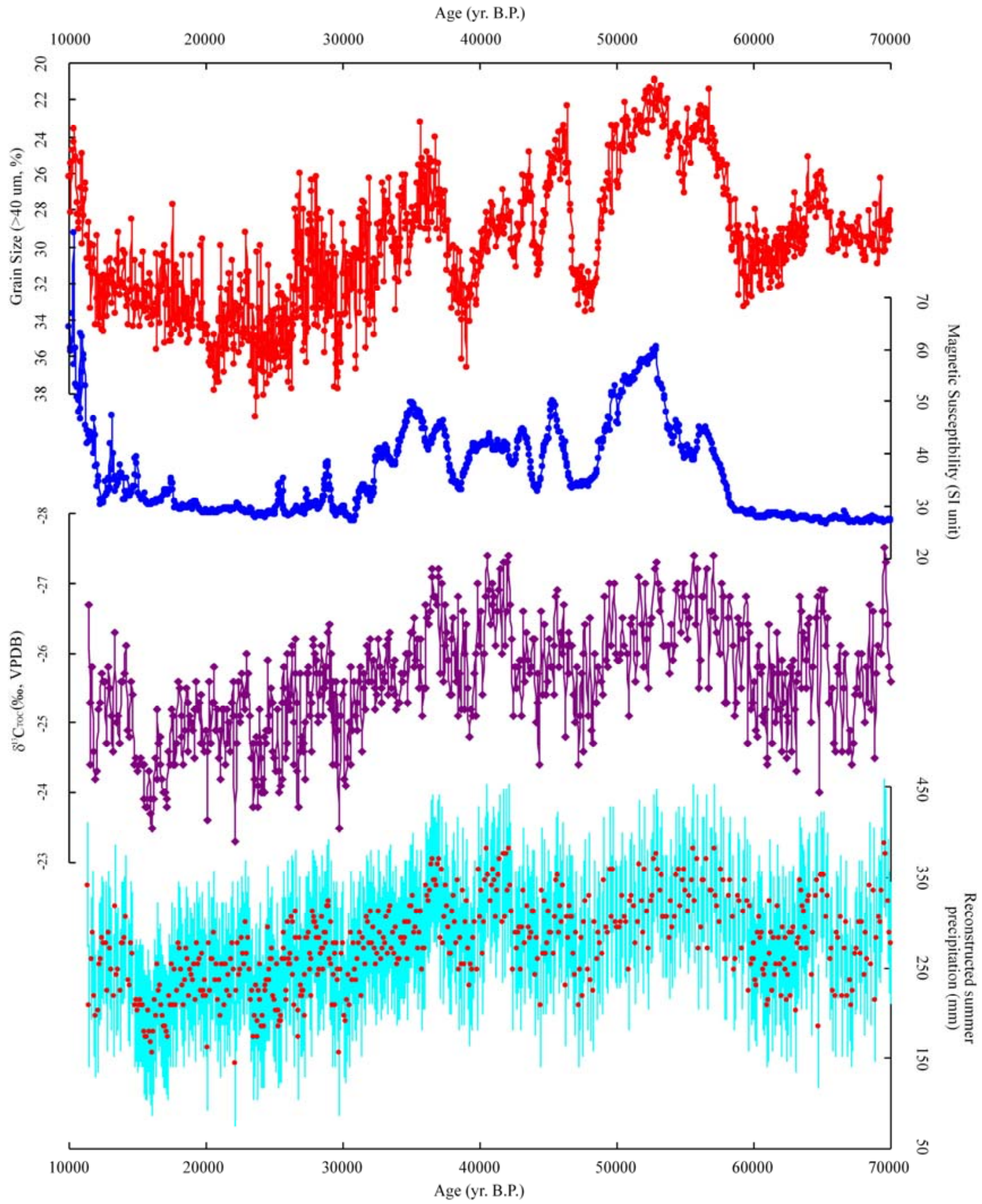

Figure S15 The original data from the YB profile plotted against the age series transferred from the NGRIP ice core. Red series are grain size data (>40μm, %); blue series are magnetic susceptibility (SI units); purple series are loess $\delta^{13}C_{TOC}$ data (‰, VPDB); calculated summer precipitation is shown by solid red dots with uncertainties represented by light blue vertical bars.

Table S2 Relevant data from the YB profile used in this paper (GS-grain size; MS-magnetic susceptibility; YD-Younger dryas event; H-Heinrich event; IS-interstadial event).

Depth （m)	OSL dating （yrs B.P.)	OSL dating errors (yrs)	NGRIP age control points	Climatic events	NGRIP ice-core ages	$\delta^{13}C_{TOC}$ (‰, VPDB)	Summer precipitation (mm)	errors (mm)	GS (>40μm, %)	MS (SI unit)
0.02						-28.0				
0.06						-27.5				
0.10						-26.3				
0.14						-26.0				
0.18						-26.4				
0.22						-25.2				
0.26						-24.8				
0.30						-26.2				
0.33	440	60								
0.34						-24.9				
0.36									25.78	106
0.38						-24.1			24.64	107
0.40									24.18	110
0.42						-24.2			25.01	112
0.44									23.55	111
0.46						-23.7			25.31	111
0.48									25.79	112
0.50						-23.8			24.32	108
0.52									26.62	111
0.54						-25.6			26.93	114
0.56									25.41	114
0.58						-24.8			26.28	116
0.60									26.22	117

0.62			-25.6	25.45	119
0.64				26.06	124
0.66	3100	200	-23.8	24.85	123
0.68				23.62	125
0.70			-25.1	23.06	137
0.72				22.87	130
0.74			-24.3	21.83	139
0.76				19.87	155
0.78			-24.3	17.03	146
0.80				18.38	158
0.82			-24.2	16.62	151
0.84				17.63	149
0.86			-24.7	16.95	153
0.88				18.45	156
0.90			-24.1	18.75	154
0.92				17.09	154
0.94			-24.3	16.03	154
0.96				15.45	158
0.98			-24.4	18.26	152
1.00	8300	600	-23.9	19.22	150
1.02				17.42	153
1.04			-25.9	17.90	151
1.06				17.09	144
1.08			-25.6	19.67	150
1.10				18.11	150
1.12			-25.8	18.31	136
1.14				17.96	147
1.16			-25.4	18.42	142
1.18				19.88	142

1.20						-25.7			20.33	142
1.22									19.72	146
1.24						-25.3			20.97	145
1.26									21.23	135
1.28						-23.4			19.75	136
1.30									21.13	140
1.32						-23.3			20.36	143
1.33	9800	700								
1.34									21.87	139
1.36						-23.6			19.97	130
1.38									20.24	130
1.40						-22.7			19.75	132
1.42									19.90	127
1.44						-23.5			20.95	130
1.46									19.57	126
1.48						-23.0			21.48	119
1.50									21.70	111
1.52						-22.6			18.71	110
1.54									20.41	119
1.56						-22.7			21.43	113
1.58									22.58	108
1.60						-24.3			22.99	112
1.62									23.91	64
1.64						-23.9			25.47	84
1.66	13700	1000							25.73	86
1.68						-23.4			24.77	93
1.70									23.51	90
1.72						-24.0			22.41	88
1.74									24.56	86

1.76				-23.9	22.36	85
1.78					23.53	84
1.80				-23.7	25.04	81
1.82					23.74	76
1.84				-24.0	24.99	69
1.86					25.51	67
1.88				-24.2	23.99	66
1.90			10024		26.20	64
1.92			10083	-24.0	25.44	60
1.94			10141		28.14	59
1.96			10200	-24.6	24.74	62
1.98			10258		25.37	67
2.00	14200	1000	10317	-24.6	24.31	82
2.02			10375		23.57	57
2.04			10434	-23.6	25.08	60
2.06			10492		25.24	53
2.08			10551	-24.4	28.23	53
2.10			10609		27.58	51
2.12			10668	-24.2	29.02	50
2.14			10726		28.69	48
2.16			10785	-24.0	27.99	63
2.18			10843		26.84	49
2.20			10902	-24.5	25.42	47
2.22			10960		24.96	55
2.24			11019	-24.5	29.83	62
2.26			11077		27.44	58
2.28			11136	-24.3	28.34	59
2.30			11194		26.86	53
2.32			11253	-24.3	26.53	46

2.33	15300	1300			11282					
2.34					11311				29.44	45
2.36					11370	-26.7	341	69	30.68	42
2.38					11428				28.74	42
2.40					11487	-24.4	208	68	31.14	44
2.42					11545				29.91	43
2.44					11604	-25.3	260	67	33.37	44
2.46					11662				31.49	43
2.48					11721	-25.8	289	68	31.41	43
2.50					11779				29.93	40
2.52					11838	-24.6	219	68	30.70	47
2.54					11896				31.50	44
2.56					11955	-24.2	196	68	34.23	38
2.58					12013				29.39	38
2.60					12072	-24.3	202	68	32.87	34
2.62			12130	YD	12130				34.30	35
2.64					12188	-25.2	254	67	31.70	36
2.66	14800	1100			12247				32.39	31
2.68					12305	-25.3	260	67	33.92	30
2.70					12364				31.85	31
2.72					12422	-25.7	283	68	34.54	31
2.74					12481				31.59	31
2.76					12539	-25.6	278	68	34.60	31
2.78					12598				32.67	32
2.80					12656	-25.6	278	68	32.73	35
2.82					12715				30.86	32
2.84					12773	-24.7	225	68	33.81	32
2.86					12832				31.74	34
2.88					12890	-25.8	289	68	32.64	35

2.90			12949				31.99	33
2.92			13007	-25.5	272	68	31.53	42
2.94			13066				30.67	33
2.96			13124	-25.1	248	67	33.07	47
2.98			13183				31.29	37
3.00	15200	1100	13241	-24.6	219	68	31.99	40
3.02			13300				31.43	35
3.04			13358	-26.3	318	68	33.68	36
3.06			13417				32.60	33
3.08			13475	-25.0	243	67	32.15	34
3.10			13534				30.73	34
3.12			13592	-25.1	248	67	29.27	34
3.14			13651				30.94	35
3.16			13709	-24.7	225	68	32.68	36
3.18			13768				31.87	38
3.20			13826	-25.6	278	68	32.11	36
3.22			13885				31.97	36
3.24			13943	-25.6	278	68	30.86	35
3.26			14002				30.19	31
3.28			14060	-25.7	283	68	30.42	31
3.30			14119				32.25	33
3.32			14177	-26.1	307	68	33.32	33
3.33	16600	1300	14206					
3.34			14236				32.60	36
3.36			14294	-24.9	237	67	34.24	33
3.38			14353				32.95	32
3.40			14411	-24.8	231	68	31.80	32
3.42			14470				32.50	33
3.44			14528	-25.6	278	68	28.55	33

3.46					14587				32.36	33
3.48					14645	-25.4	266	67	33.16	34
3.50					14704				34.34	33
3.52					14762	-24.4	208	68	33.38	34
3.54					14821				30.80	36
3.56					14879	-24.5	214	68	33.06	39
3.58					14938				32.20	38
3.60					14996	-24.3	202	68	33.35	39
3.62					15055				32.37	36
3.64					15113	-24.4	208	68	33.10	33
3.66	16900	1300			15172				34.35	32
3.68			15230	H1	15230	-24.5	214	68	34.32	32
3.70					15278				33.25	31
3.72					15325	-24.4	208	68	31.07	32
3.74					15373				32.38	32
3.76					15421	-24.4	208	68	30.31	32
3.78					15468				33.20	32
3.80					15516	-23.9	179	69	33.51	33
3.82					15564				32.99	31
3.84					15611	-23.8	173	69	32.56	31
3.86					15659				31.98	31
3.88					15707	-23.8	173	69	32.68	31
3.90					15755				33.35	31
3.92					15802	-24.3	202	68	33.87	31
3.94					15850				32.75	30
3.96					15898	-23.9	179	69	31.45	31
3.98					15945				31.94	31
4.00	17300	1300			15993	-23.7	167	69	34.30	31
4.02					16041				32.15	31

4.04			16088	-23.5	156	69	33.84	31
4.06			16136				33.61	31
4.08			16184	-23.9	179	69	33.76	31
4.10			16231				33.44	31
4.12			16279	-24.5	214	68		31
4.14			16327				33.06	31
4.16			16374	-24.2	196	68	35.62	31
4.18			16422				30.40	31
4.20			16470	-25.2	254	67	32.86	31
4.22	17500	1200	16517	-24.7	225	68	33.70	31
4.24			16565				32.20	31
4.26			16613	-24.8	231	68	34.20	31
4.28			16661				32.44	31
4.30			16708	-24.4	208	68	30.52	32
4.32			16756				32.44	31
4.34			16804	-24.2	196	68	32.23	31
4.36			16851				30.42	32
4.38			16899	-24.0	185	68	32.09	32
4.40			16947				31.72	33
4.42			16994	-24.0	185	68	35.25	33
4.44			17042				29.96	33
4.46			17090	-23.9	179	69	33.12	33
4.48			17137				33.18	33
4.50			17185	-23.8	173	69	32.88	32
4.52			17233				34.26	33
4.54			17280	-24.6	219	68	30.87	33
4.56			17328			68	34.20	33
4.58			17376	-24.4	208	68	35.18	33
4.60			17423				35.20	35

4.62					17471	-25.2	254	67	34.81	34
4.64					17519				32.34	35
4.66					17566	-24.4	208	68	27.67	34
4.68					17614				33.96	33
4.70					17662	-25.1	248	67	31.34	31
4.72	18200	1300			17710				32.22	31
4.74					17757	-24.4	208	68	34.53	30
4.76					17805				33.72	30
4.78					17853	-24.7	225	68	31.06	30
4.80					17900				33.77	30
4.82					17948	-25.6	278	68	31.51	30
4.84					17996				34.01	30
4.86					18043	-25.5	272	68	33.50	30
4.88					18091				34.30	30
4.90					18139	-25.1	248	67	34.77	29
4.92					18186				32.04	29
4.94					18234	-24.8	231	68	32.06	30
4.96					18282				33.68	30
4.98					18329	-24.4	208	68	30.54	30
5.00					18377				33.93	30
5.02					18425	-24.9	237	67	33.95	30
5.04					18472				33.97	30
5.06					18520	-25.5	272	68	33.34	30
5.08					18568				33.13	30
5.10					18616	-25.3	260	67	35.26	30
5.12					18663				34.51	30
5.14					18711	-24.6	219	68	32.79	30
5.16					18759				33.11	30
5.18					18806	-25.1	248	67	35.07	30

5.20			18854				33.13	30
5.22			18902	-25.0	243	67	34.30	30
5.24			18949				30.52	31
5.26			18997	-24.9	237	67	33.98	30
5.28			19045				34.03	30
5.30			19092	-25.2	254	67	34.32	30
5.32			19140				32.52	30
5.34			19188	-24.5	214	68	32.03	31
5.36			19235				32.65	31
5.38			19283	-25.6	278	68	32.46	31
5.40			19331				32.03	30
5.42			19378	-25.2	254	67	32.11	30
5.44			19426				31.91	30
5.46			19474	-25.3	260	67	33.75	30
5.48			19522				34.01	30
5.50			19569	-24.7	225	68	34.38	29
5.52			19617				34.40	29
5.54			19665	-25.4	266	67	29.92	29
5.56			19712				33.09	29
5.58			19760	-24.7	225	68	29.64	29
5.60			19808				35.16	29
5.62			19855	-24.6	219	68	34.32	29
5.64			19903				34.28	29
5.66			19951	-24.9	237	67	34.26	29
5.68			19998				34.31	29
5.70			20046	-24.6	219	68	34.77	29
5.72	19300	1400	20094				34.83	29
5.74			20141	-23.6	161	69	34.32	29
5.76			20189				36.36	29

5.78					20237	-25.6	278	68	35.65	29
5.80					20284				35.36	29
5.82					20332	-24.7	225	68	36.52	29
5.84					20380				36.08	29
5.86					20428	-25.3	260	67	35.53	29
5.88					20475				36.18	29
5.90					20523	-25.3	260	67	36.20	29
5.92					20571				34.81	29
5.94					20618	-25.8	289	68	37.83	29
5.96					20666				36.71	29
5.98					20714	-24.9	237	67	35.36	29
6.00					20761				37.15	29
6.02					20809	-25.2	254	67	37.39	29
6.04					20857				36.41	29
6.06					20904	-24.5	214	68	34.05	29
6.08					20952				37.43	29
6.10					21000	-24.9	237	67	33.86	29
6.12					21047				32.24	29
6.14					21095	-24.2	196	68	35.51	29
6.16					21143				29.99	29
6.18					21190	-25.2	254	67	35.91	29
6.20					21238				34.64	29
6.22					21286	-25.2	254	67	33.17	29
6.24					21334				34.66	30
6.26					21381	-24.4	208	68	36.87	30
6.28					21429				33.88	30
6.30					21477	-25.2	254	67	35.30	29
6.32					21524				32.42	30
6.34					21572	-24.7	225	68	35.62	29

6.36			21620				34.09	29
6.38			21667	-25.0	243	67	31.46	29
6.40			21715				33.93	30
6.42			21763	-24.6	219	68	33.65	29
6.44			21810				32.01	29
6.46			21858	-25.3	260	67	34.79	29
6.48			21906				34.33	30
6.50			21953	-25.6	278	68	30.80	30
6.52			22001				35.33	30
6.54			22049	-25.1	248	67	36.42	30
6.56			22096				35.61	30
6.58			22144	-23.3	144	70	33.07	30
6.60			22192				33.44	30
6.62			22239	-24.7	225	68	33.49	30
6.64			22287				34.62	30
6.66			22335	-25.6	278	68	34.24	31
6.68			22383				33.31	30
6.70			22430	-25.0	243	67	35.83	30
6.72	20200	1900	22478				32.41	29
6.74			22526	-25.1	248	67	33.32	29
6.76			22573				31.57	30
6.78			22621	-25.7	283	68	35.35	29
6.80			22669				34.70	29
6.82			22716	-25.4	266	67	32.88	29
6.84			22764				35.01	29
6.86			22812	-25.2	254	67	34.46	29
6.88			22859				31.96	29
6.90			22907	-26.0	301	68	29.27	29
6.92			22955				31.42	29

6.94					23002	-25.4	266	67	34.71	29
6.96					23050				33.00	29
6.98					23098	-25.7	283	68	31.37	29
7.00					23145				35.17	29
7.02					23193	-25.1	248	67	34.80	30
7.04					23241				34.87	29
7.06					23289	-24.5	214	68	36.48	30
7.08					23336				36.32	29
7.10					23384	-24.7	225	68	37.36	29
7.12					23432				33.30	29
7.14					23479	-23.8	173	69	34.42	28
7.16					23527				37.52	28
7.18					23575	-24.2	196	68	37.24	29
7.20					23622				35.32	29
7.22			23670	H2	23670	-24.8	231	68	39.32	28
7.24					23701				34.86	29
7.26					23732	-24.1	190	68	38.21	28
7.28					23762				30.27	28
7.30					23793	-23.8	173	69	35.01	29
7.32					23824				33.37	29
7.34					23855	-24.7	225	68	35.08	29
7.36					23886				35.96	29
7.38					23917	-25.2	254	67	34.22	28
7.40					23947				32.12	29
7.42					23978	-24.5	214	68	36.72	28
7.44					24009				29.97	28
7.46					24040	-24.0	185	68	36.80	28
7.48					24071				31.99	28
7.50					24102	-24.0	185	68	35.73	29

7.52				24132				35.59	28
7.54				24163	-24.2	196	68	36.82	28
7.56				24194				38.14	28
7.58				24225	-24.0	185	68	35.09	28
7.60				24256				34.76	28
7.62				24286	-24.8	231	68	35.98	28
7.64				24317				35.73	28
7.66				24348	-24.7	225	68	35.52	28
7.68				24379				34.84	28
7.70				24410	-24.9	237	67	34.76	28
7.72	21400	1700		24441				34.05	28
7.74				24471	-24.9	237	67	37.51	29
7.76				24502				35.07	29
7.78				24533	-25.9	295	68	33.95	29
7.80				24564				36.17	28
7.82				24595	-24.9	237	67	35.24	29
7.84				24626				31.02	29
7.86				24656	-25.1	248	67	35.51	29
7.88				24687				35.92	29
7.90				24718	-25.3	260	67	37.08	29
7.92				24749				34.58	29
7.94				24780	-25.5	272	68	33.52	29
7.96				24811				36.24	29
7.98				24841	-25.3	260	67	35.53	29
8.00				24872				35.36	29
8.02				24903	-24.6	219	68	35.34	29
8.04				24934				35.42	28
8.06				24965	-24.5	214	68	33.85	29
8.08				24995				35.71	29

8.10					25026	-24.5	214	68	36.78	29
8.12					25057				35.87	29
8.14					25088	-24.3	202	68	33.42	29
8.16					25119				36.10	30
8.18					25150	-25.2	254	67	35.96	30
8.20					25180				35.74	30
8.22					25211	-25.2	254	67	35.21	30
8.24					25242				34.28	31
8.26					25273	-24.3	202	68	32.24	32
8.28					25304				33.39	34
8.30					25335	-24.0	185	68	32.67	34
8.32					25365				36.08	34
8.34					25396	-24.1	190	68	33.95	33
8.36					25427				35.29	32
8.38					25458	-24.1	190	68	36.61	31
8.40					25489				34.55	31
8.42					25519	-24.2	196	68	32.54	31
8.44					25550				35.08	31
8.46					25581	-25.3	260	67	34.60	31
8.48					25612				35.72	33
8.50					25643	-25.8	289	68	33.36	36
8.52					25674				32.71	30
8.54					25704	-25.5	272	68	33.96	30
8.56					25735				33.97	29
8.58					25766	-25.3	260	67	35.34	29
8.60					25797				36.03	29
8.62					25828	-24.5	214	68	33.68	28
8.64					25859				32.90	28
8.66					25889	-24.7	225	68	34.24	29

8.68			25920				35.24	29
8.70			25951	-26.0	301	68	33.88	28
8.72	21400	1600	25982				37.42	28
8.74			26013	-25.2	254	67	34.72	29
8.76			26044				35.74	29
8.78			26074	-25.3	260	67	36.59	29
8.80			26105				34.67	29
8.82			26136	-25.4	266	67	36.37	29
8.84			26167				36.43	28
8.86			26198	-25.2	254	67	31.67	28
8.88			26228				37.78	28
8.90			26259	-26.1	307	68	35.09	28
8.92			26290				33.98	28
8.94			26321	-26.0	301	68	34.05	28
8.96			26352				33.16	29
8.98			26383	-25.0	243	67	34.85	29
9.00			26413				32.18	29
9.02			26444	-24.4	208	68	34.30	29
9.04			26475				30.32	29
9.06			26506	-26.2	312	68	32.42	29
9.08			26537				28.03	29
9.10			26568	-25.7	283	68	33.57	29
9.12			26598				33.93	29
9.14			26629	-25.3	260	67	30.71	29
9.16			26660				28.44	30
9.18			26691	-24.3	202	68	29.34	29
9.20			26722				30.95	29
9.22			26752	-23.8	173	69	27.31	29
9.24			26783				28.72	29

9.26					26814	-25.7	283	68	26.03	29
9.28					26845				30.36	29
9.30					26876	-24.6	219	68	32.34	29
9.32					26907				29.48	29
9.34					26937	-24.8	231	68	32.72	29
9.36					26968				33.33	29
9.38					26999	-24.7	225	68	29.21	29
9.40					27030				33.82	29
9.42					27061	-24.6	219	68	32.30	30
9.44					27092				35.74	29
9.46					27122	-24.7	225	68	27.97	29
9.48					27153				33.94	29
9.50					27184	-25.0	243	67	35.29	29
9.52					27215				31.85	29
9.54					27246	-24.2	196	68	32.30	29
9.56					27276				34.27	29
9.58					27307	-25.7	283	68	33.79	29
9.60					27338				34.75	30
9.62					27369	-25.7	283	68	36.35	32
9.64					27400				31.18	33
9.66					27431	-25.7	283	68	34.40	32
9.68					27461				29.14	30
9.70					27492	-25.8	289	68	29.30	30
9.72	21700	1700			27523				31.32	31
9.74					27554	-25.1	248	67	29.11	30
9.76					27585				28.65	31
9.78					27616	-25.4	266	67	28.23	30
9.80					27646				30.78	30
9.82					27677	-25.3	260	67	26.36	31

9.84	27708				27.04	31
9.86	27739	-24.6	219	68	31.51	31
9.88	27770				28.64	31
9.90	27801	-26.2	312	68	29.21	31
9.92	27831				33.39	30
9.94	27862	-26.1	307	68	30.53	30
9.96	27893				31.52	30
9.98	27924	-26.0	301	68	26.40	30
10.00	27955				32.22	31
10.02	27985	-26.0	301	68	34.28	31
10.04	28016				28.24	30
10.06	28047	-25.7	283	68	33.73	31
10.08	28078				26.16	30
10.10	28109	-25.9	295	68	30.32	30
10.12	28140				31.62	30
10.14	28170	-25.0	243	67	28.02	29
10.16	28201				34.38	30
10.18	28232	-25.1	248	67	32.38	29
10.20	28263				31.86	30
10.22	28294	-25.7	283	68	30.33	30
10.24	28325				31.89	30
10.26	28355	-25.4	266	67	32.84	30
10.28	28386				28.49	31
10.30	28417	-26.1	307	68	32.95	31
10.32	28448				32.10	30
10.34	28479	-25.5	272	68	33.32	31
10.36	28509				33.00	31
10.38	28540	-25.1	248	67	31.68	31
10.40	28571				31.47	31

10.42					28602	-25.5	272	68	29.89	32
10.44					28633				34.62	32
10.46					28664	-25.8	289	68	29.39	33
10.48					28694				35.75	34
10.50					28725	-25.8	289	68	31.89	35
10.52					28756				30.96	36
10.54					28787	-25.6	278	68	34.05	36
10.56					28818				32.11	38
10.58					28849	-26.3	318	68	34.02	38
10.60					28879				33.12	39
10.62					28910	-26.3	318	68	33.28	38
10.64					28941				30.37	38
10.66					28972	-26.4	324	69	33.59	38
10.68					29003				32.35	36
10.70					29034	-25.3	260	67	30.86	34
10.72	24500	1900			29064				32.09	33
10.74					29095	-25.2	254	67	33.25	33
10.76					29126				33.78	33
10.78					29157	-26.0	301	68	31.16	31
10.80					29188				28.14	31
10.82					29218	-24.4	208	68	31.37	30
10.84					29249				30.05	30
10.86					29280	-25.6	278	68	33.30	29
10.88					29311				32.64	30
10.90					29342	-25.0	243	67	31.19	29
10.92					29373				32.57	30
10.94					29403	-25.4	266	67	34.92	29
10.96					29434				37.66	29
10.98					29465	-24.4	208	68	36.41	29

11.00				29496				30.38	29
11.02				29527	-25.6	278	68	31.65	28
11.04				29558				35.59	28
11.06				29588	-25.1	248	67	36.85	29
11.08				29619				37.16	29
11.10		29650	H3	29650	-24.9	237	67	37.72	29
11.12				29697				34.83	29
11.14				29745	-23.5	156	69	33.73	29
11.16				29792				35.26	29
11.18				29839	-25.1	248	67	36.29	29
11.20				29886				35.30	29
11.22				29934	-25.4	266	67	34.21	30
11.24				29981				28.83	30
11.26				30028	-25.6	278	68	31.48	30
11.28				30075				29.74	30
11.30				30123	-24.2	196	68	33.70	30
11.32				30170				32.74	29
11.34				30217	-24.1	190	68	30.42	29
11.36				30265				33.65	28
11.38				30312	-24.5	214	68	31.19	28
11.40				30359				32.78	28
11.42				30406	-25.0	243	67	31.50	28
11.44				30454				34.39	28
11.46				30501	-24.4	208	68	30.88	28
11.48				30548				32.39	27
11.50				30595	-25.8	289	68	31.27	27
11.52				30643				30.45	27
11.54				30690	-24.9	237	67	30.57	27
11.56				30737				32.02	27

11.58					30785	-25.6	278	68	32.27	28
11.60					30832				32.03	27
11.62					30879	-25.1	248	67	30.15	27
11.64					30926				36.72	28
11.66					30974	-24.9	237	67	35.94	30
11.68					31021				30.28	31
11.70					31068	-25.3	260	67	30.44	30
11.72					31115				30.21	31
11.74					31163	-25.6	278	68	30.66	32
11.76					31210				33.62	33
11.78					31257	-25.8	289	68	28.44	33
11.80					31305				32.45	33
11.82					31352	-24.6	219	68	27.97	33
11.84					31399				30.42	34
11.86					31446	-25.7	283	68	27.51	34
11.88					31494				30.74	34
11.90					31541	-25.5	272	68	29.39	34
11.92					31588				27.58	33
11.94					31635	-25.2	254	67	28.15	33
11.96					31683				35.47	33
11.98					31730	-26.1	307	68	33.99	33
12.00					31777				32.45	32
12.02					31825	-26.0	301	68	32.81	33
12.04					31872				26.27	33
12.06					31919	-25.6	278	68	34.29	33
12.08					31966				28.75	33
12.10					32014	-26.2	312	68	33.23	33
12.12					32061				31.37	31
12.14					32108	-25.6	278	68	33.28	31

12.16			32155				32.61	32
12.18			32203	-25.9	295	68	31.08	32
12.20			32250				34.77	33
12.22	24200	1800	32297	-25.5	272	68	33.96	34
12.24			32345				30.67	35
12.26			32392	-25.2	254	67	31.96	39
12.28			32439				30.97	39
12.30			32486	-25.4	266	67	32.25	39
12.32			32534				28.80	39
12.34			32581	-26.2	312	68	30.98	41
12.36			32628				29.49	41
12.38			32675	-25.8	289	68	29.57	41
12.40			32723				29.27	41
12.42			32770	-25.3	260	67	28.73	41
12.44			32817				30.48	40
12.46			32865	-25.5	272	68	28.78	39
12.48			32912				26.54	40
12.50			32959	-25.7	283	68	28.57	40
12.52			33006				31.95	40
12.54			33054	-25.6	278	68	26.97	40
12.56			33101				29.56	41
12.58			33148	-26.1	307	68	28.25	42
12.60			33195				27.80	41
12.62			33243	-25.4	266	67	27.56	40
12.64			33290				28.16	40
12.66			33337	-26.2	312	68	27.75	39
12.68			33385				26.27	39
12.70			33432	-26.3	318	68	26.26	39
12.72			33479				29.25	39

12.74					33526	-25.5	272	68	28.66	38
12.76					33574				28.45	38
12.78					33621	-25.8	289	68	30.01	39
12.80					33668				29.08	39
12.82	26100	1900			33715	-25.4	266	67	29.23	38
12.84					33763				30.30	39
12.86					33810	-25.9	295	68	31.77	38
12.88					33857				33.47	38
12.90					33905	-25.2	254	67	29.71	38
12.92					33952				29.56	40
12.94					33999	-25.3	260	67	30.50	40
12.96					34046				30.85	40
12.98					34094	-25.3	260	67	27.27	41
13.00					34141				31.94	42
13.02					34188	-25.7	283	68	30.07	42
13.04					34235				29.56	42
13.06					34283	-25.7	283	68	26.77	43
13.08					34330				26.09	43
13.10					34377	-25.6	278	68	27.50	44
13.12					34425				27.85	44
13.14					34472	-25.7	283	68	27.15	44
13.16					34519				26.13	45
13.18					34566	-26.0	301	68	29.07	45
13.20					34614				28.69	46
13.22					34661	-26.0	301	68	28.25	46
13.24					34708				28.28	47
13.26					34755	-25.3	260	67	30.88	48
13.28					34803				30.84	47
13.30					34850	-26.0	301	68	31.45	48

13.32				34897				29.63	48
13.34				34945	-26.3	318	68	28.74	48
13.36				34992				29.94	50
13.38				35039	-26.3	318	68	29.24	50
13.40				35086				27.85	50
13.42				35134	-26.5	330	69	29.00	49
13.44				35181				28.24	49
13.46				35228	-25.8	289	68	28.29	49
13.48				35275				28.48	48
13.50				35323	-25.9	295	68	28.60	47
13.52				35370				27.82	47
13.54				35417	-26.2	312	68	26.57	47
13.56				35465				28.64	48
13.58				35512	-25.5	272	68	25.57	48
13.60				35559				28.24	48
13.62				35606	-25.8	289	68	28.95	48
13.64				35654				23.23	47
13.66				35701	-26.2	312	68	26.74	47
13.68				35748				25.22	47
13.70				35795	-25.1	248	67	27.59	46
13.72	30000	2500		35843				27.23	45
13.74				35890	-25.5	272	68	26.23	46
13.76				35937				25.56	45
13.78				35985	-25.5	272	68	29.08	45
13.80				36032				26.07	43
13.82				36079	-26.7	341	69	27.36	42
13.84				36126				28.66	41
13.86				36174	-26.5	330	69	24.83	41
13.88				36221				29.71	41

13.90					36268	-26.4	324	69	25.77	41
13.92					36315				29.08	42
13.94					36363	-26.6	336	69	25.17	42
13.96					36410				25.44	42
13.98					36457	-27.1	365	70	27.98	42
14.00					36505				27.97	43
14.02					36552	-27.2	370	70	28.67	43
14.04					36599				25.92	43
14.06					36646	-26.8	347	69	27.78	43
14.08					36694				25.37	44
14.10					36741	-26.8	347	69	24.03	44
14.12					36788				25.92	44
14.14					36835	-26.7	341	69	27.69	45
14.16					36883				29.10	45
14.18					36930	-27.1	365	70	25.50	45
14.20					36977				26.91	45
14.22					37025	-27.2	370	70	27.26	45
14.24					37072				28.19	46
14.26					37119	-25.7	283	68	26.92	46
14.28					37166				29.58	46
14.30					37214	-27.0	359	70	27.88	45
14.32					37261				27.16	46
14.34					37308	-25.6	278	68	27.65	45
14.36					37355					46
14.38					37403	-26.1	307	68	28.80	45
14.40					37450				26.98	44
14.42					37497	-26.7	341	69	29.14	44
14.44					37545				28.98	43
14.46					37592	-26.3	318	68	30.47	42

14.48	37639				31.32	41
14.50	37686	-25.9	295	68	28.62	41
14.52	37734				30.48	39
14.54	37781	-25.4	266	67	30.17	38
14.56	37828					38
14.58	37875	-25.4	266	67	32.38	37
14.60	37923				31.49	37
14.62	37970	-26.2	312	68	33.36	35
14.64	38017				32.31	36
14.66	38065	-26.5	330	69	29.99	36
14.68	38112				32.33	35
14.70	38159	-26.0	301	68	31.29	35
14.72	38206				31.12	34
14.74	38254	-25.6	278	68	32.88	35
14.76	38301				32.57	35
14.78	38348	-26.8	347	69	30.11	34
14.80	38395				32.66	34
14.82	38443	-25.1	248	67	33.66	34
14.84	38490				32.73	33
14.86	38537	-25.7	283	68	33.06	33
14.88	38585				30.34	33
14.90	38632	-25.2	254	67	33.98	33
14.92	38679				36.13	34
14.94	38726	-26.6	336	69	31.32	34
14.96	38774				33.05	36
14.98	38821	-25.2	254	67	32.10	36
15.00	38868				33.59	36
15.02	38915	-26.0	301	68	33.18	36
15.04	38963				33.23	37

15.06			39010	H4	39010	-26.4	324	69	36.57	37
15.08					39064				33.30	38
15.10					39117	-25.5	272	68	32.30	38
15.12					39171				32.82	39
15.14					39225	-24.8	231	68	32.87	39
15.16					39279				34.08	39
15.18					39332	-25.2	254	67	32.45	40
15.20					39386				32.12	40
15.22	33800	2800			39440	-25.1	248	67	32.62	40
15.24					39493				30.78	41
15.26					39547	-25.7	283	68	30.22	42
15.28					39601				31.62	42
15.30					39654	-26.0	301	68	30.89	42
15.32					39708				33.20	41
15.34					39762	-25.1	248	67	32.10	41
15.36					39816				32.94	40
15.38					39869	-27.0	359	70	31.36	41
15.40					39923				30.37	42
15.42					39977	-26.1	307	68	30.86	41
15.44					40030				30.46	41
15.46					40084	-26.6	336	69	29.20	42
15.48					40138				31.15	42
15.50					40191	-25.4	266	67	30.14	42
15.52					40245				29.64	42
15.54					40299	-26.0	301	68	29.68	42
15.56					40353				29.88	42
15.58					40406	-26.8	347	69	29.10	42
15.60					40460				28.12	42
15.62					40514	-27.4	382	71	29.51	42

15.64				40567				30.29	43
15.66				40621	-26.9	353	70	30.20	43
15.68				40675				29.20	43
15.70				40729	-26.4	324	69	29.33	44
15.72	35900	2800		40782				28.63	43
15.74				40836	-26.7	341	69	27.66	42
15.76				40890				28.79	42
15.78				40943	-27.0	359	70	28.61	41
15.80				40997				27.82	41
15.82				41051	-26.8	347	69	29.00	41
15.84				41104				29.00	41
15.86				41158	-26.1	307	68	29.18	41
15.88				41212				30.03	41
15.90				41266	-26.6	336	69	29.32	42
15.92				41319				29.42	42
15.94				41373	-26.9	353	70	29.00	42
15.96				41427				28.40	41
15.98				41480	-27.2	370	70	28.50	41
16.00				41534				29.19	41
16.02				41588	-26.0	301	68	29.25	41
16.04				41641				26.93	40
16.06				41695	-27.3	376	71	28.34	42
16.08				41749				28.97	42
16.10				41803	-26.1	307	68	29.32	42
16.12				41856				28.41	42
16.14				41910	-27.3	376	71	28.55	42
16.16				41964				27.63	41
16.18				42017	-26.6	336	69	27.51	42
16.20				42071				27.54	41

16.22					42125	-27.4	382	71	28.32	39
16.24					42179				28.58	39
16.26					42232	-26.7	341	69	28.14	38
16.28					42286				29.60	38
16.30					42340	-26.2	312	68	30.21	38
16.32					42393				30.41	38
16.34					42447	-25.1	248	67	30.40	38
16.36					42501				30.12	38
16.38					42554	-25.8	289	68	30.57	39
16.40					42608				29.54	39
16.42					42662	-25.5	272	68	31.11	40
16.44					42716				28.77	41
16.46					42769	-25.8	289	68	29.59	42
16.48					42823				29.65	43
16.50					42877	-25.9	295	68	28.94	43
16.52					42930				29.38	43
16.54					42984	-25.1	248	67	28.85	44
16.56					43038				27.62	45
16.58					43091	-25.9	295	68	28.77	45
16.60					43145				26.12	44
16.62					43199	-26.6	336	69	27.71	44
16.64					43253				27.68	44
16.66					43306	-25.6	278	68	26.89	44
16.68					43360				27.51	43
16.70					43414	-26.3	318	68	26.21	43
16.72	35400	3100			43467				27.33	43
16.74					43521	-25.9	295	68	27.31	42
16.76					43575				24.85	41
16.78					43629	-25.7	283	68	26.54	40

16.80	43682				26.20	39
16.82	43736	-25.8	289	68	27.10	37
16.84	43790				27.51	36
16.86	43843	-26.2	312	68	27.28	36
16.88	43897				29.96	35
16.90	43951	-26.2	312	68	28.77	34
16.92	44004				29.81	34
16.94	44058	-25.0	243	67	30.37	34
16.96	44112				29.99	34
16.98	44166	-25.7	283	68	30.01	33
17.00	44219				30.92	33
17.02	44273	-25.3	260	67	31.60	33
17.04	44327				31.28	34
17.06	44380	-24.4	208	68	30.68	34
17.08	44434				30.12	35
17.10	44488	-26.6	336	69	30.92	35
17.12	44541				28.87	38
17.14	44595	-25.4	266	67	29.21	39
17.16	44649				28.66	40
17.18	44703	-26.4	324	69	28.04	41
17.20	44756				28.89	42
17.22	44810	-25.4	266	67	28.28	42
17.24	44864				26.80	42
17.26	44917	-25.6	278	68	26.53	43
17.28	44971				27.24	43
17.30	45025	-26.2	312	68	24.98	43
17.32	45079				26.88	45
17.34	45132	-25.8	289	68	25.63	47
17.36	45186				26.54	48

17.38					45240	-25.8	289	68	26.76	49
17.40					45293				25.52	50
17.42					45347	-25.4	266	67	25.14	50
17.44					45401				26.03	49
17.46					45454	-26.1	307	68	25.75	50
17.48					45508				24.77	49
17.50					45562	-26.8	347	69	24.18	47
17.52					45616				24.80	46
17.54					45669	-26.9	353	70	24.83	45
17.56					45723				24.95	44
17.58					45777	-25.8	289	68	23.73	44
17.60					45830				25.32	44
17.62					45884	-26.3	318	68	25.17	44
17.64					45938				26.39	44
17.66					45991	-26.0	301	68	23.68	43
17.68	36900	3000			46045				24.84	42
17.70					46099	-26.7	341	69	23.41	41
17.72					46153				24.74	41
17.74					46206	-24.8	231	68	23.79	40
17.76					46260				25.51	42
17.78					46314	-26.1	307	68	25.99	39
17.80					46367				22.36	38
17.82					46421	-25.7	283	68	25.86	38
17.84					46475				25.44	36
17.86					46529	-26.3	318	68	26.55	36
17.88					46582				28.08	35
17.90					46636	-26.1	307	68	27.75	34
17.92					46690				29.49	34
17.94					46743	-26.1	307	68	30.19	33

17.96			46797				31.18	34
17.98			46851	-25.4	266	67	31.52	34
18.00			46904				31.39	34
18.02			46958	-25.0	243	67	31.53	34
18.04			47012				32.34	34
18.06			47066	-25.8	289	68	31.54	34
18.08			47119				31.29	34
18.10			47173	-24.4	208	68	31.81	34
18.12			47227				33.22	34
18.14			47280	-25.1	248	67	32.26	34
18.16			47334				31.44	34
18.18			47388	-25.7	283	68	32.08	34
18.20			47441				31.10	34
18.22			47495	-24.6	219	68	32.00	35
18.24			47549				31.71	34
18.26			47603	-26.0	301	68	32.25	35
18.28			47656				32.53	34
18.30	47710	H5	47710	-26.4	324	69	33.54	34
18.32			47778				32.02	35
18.34			47846	-25.1	248	67	33.06	34
18.36			47913				32.35	34
18.38			47981	-26.5	330	69	31.38	35
18.40			48049				32.77	35
18.42			48117	-24.9	237	67	32.36	35
18.44			48185				32.03	35
18.46			48253	-24.7	225	68	33.42	35
18.48			48320				32.19	35
18.50			48388	-26.0	301	68	32.02	36
18.52			48456				31.90	36

18.54					48524	-25.3	260	67	30.98	36
18.56					48592				31.71	37
18.58					48659	-25.8	289	68	30.11	39
18.60					48727				29.76	42
18.62					48795	-25.6	278	68	27.50	43
18.64					48863				28.72	42
18.66					48931	-25.4	266	67	29.09	41
18.68					48999				28.04	42
18.70					49066	-26.8	347	69	26.88	43
18.72	42300	3300			49134				28.09	44
18.74					49202	-25.9	295	68	27.35	45
18.76					49270				26.45	45
18.78					49338	-25.8	289	68	26.00	47
18.80					49405				24.44	45
18.82					49473	-27.0	359	70	26.95	44
18.84					49541				26.71	47
18.86					49609	-26.1	307	68	28.12	52
18.88					49677				23.39	51
18.90					49744	-27.0	359	70	24.63	51
18.92					49812				24.37	51
18.94					49880	-26.0	301	68	23.64	53
18.96					49948				23.40	46
18.98					50016	-25.9	295	68	26.48	46
19.00					50084				26.65	46
19.02					50151	-25.9	295	68	26.77	48
19.04					50219				25.93	51
19.06					50287	-26.0	301	68	24.64	51
19.08					50355				24.44	52
19.10					50423	-26.5	330	69	23.12	52

19.12	50490				24.64	54
19.14	50558	-26.1	307	68	24.85	54
19.16	50626				22.17	55
19.18	50694	-26.0	301	68	23.49	55
19.20	50762				22.95	54
19.22	50830	-25.1	248	67	23.14	53
19.24	50897				23.42	54
19.26	50965	-26.4	324	69	24.52	53
19.28	51033				23.91	54
19.30	51101	-26.5	330	69	23.95	54
19.32	51169				24.77	54
19.34	51236	-26.3	318	68	24.63	54
19.36	51304				22.64	54
19.38	51372	-25.6	278	68	23.96	55
19.40	51440				23.17	55
19.42	51508	-26.0	301	68	23.39	56
19.44	51576				23.25	56
19.46	51643	-27.1	365	70	23.07	57
19.48	51711				22.85	57
19.50	51779	-26.4	324	69	23.17	58
19.52	51847				23.70	58
19.54	51915	-25.8	289	68	23.76	57
19.56	51982				23.24	58
19.58	52050	-27.0	359	70	22.00	58
19.60	52118				21.73	58
19.62	52186	-26.2	312	68	21.55	58
19.64	52254				23.15	57
19.66	52321	-25.5	272	68	23.84	58
19.68	52389				22.11	59

19.70					52457	-26.4	324	69	21.31	58
19.72	47100	4100			52525				21.40	58
19.74					52593	-26.5	330	69	22.23	59
19.76					52661				23.13	60
19.78					52728	-27.2	370	70	20.90	59
19.80					52796				21.01	60
19.82					52864	-27.3	376	71	22.39	60
19.84					52932				21.99	60
19.86					53000	-27.0	359	70	21.51	54
19.88					53067				22.59	54
19.90					53135	-26.6	336	69	21.64	53
19.92					53203				22.02	53
19.94					53271	-26.9	353	70	21.29	53
19.96					53339				23.49	52
19.98					53407	-26.1	307	68	22.83	52
20.00					53474				23.12	51
20.02					53542	-26.1	307	68	23.43	50
20.04					53610				22.21	48
20.06					53678	-26.1	307	68	21.98	45
20.08					53746				25.11	45
20.10					53813	-25.7	283	68	24.29	45
20.12					53881				24.75	44
20.14					53949	-26.4	324	69	24.35	45
20.16					54017				23.82	44
20.18					54085	-25.9	295	68	24.09	43
20.20					54153				23.72	42
20.22					54220	-26.1	307	68	23.88	43
20.24					54288				23.53	45
20.26					54356	-26.9	353	70	23.40	46

20.28				54424				23.30	46
20.30				54492	-27.0	359	70	23.89	45
20.32				54559				24.06	46
20.34				54627	-26.3	318	68	25.97	44
20.36				54695				24.66	41
20.38				54763	-26.8	347	69	24.98	41
20.40				54831				26.43	40
20.42				54899	-27.0	359	70	27.08	40
20.44				54966				26.10	39
20.46				55034	-26.6	336	69	24.93	39
20.48				55102				24.32	40
20.50				55170	-26.2	312	68	22.47	42
20.52				55238				24.10	41
20.54				55305	-26.5	330	69	24.49	40
20.56				55373				25.48	40
20.58				55441	-26.8	347	69	24.15	41
20.60				55509				23.56	39
20.62				55577	-27.4	382	71	23.60	39
20.64				55644				24.03	39
20.66				55712	-26.4	324	69	23.88	40
20.68				55780				23.43	40
20.70				55848	-27.2	370	70	23.65	41
20.72	51200	4600		55916				23.49	42
20.74				55984	-25.5	272	68	22.75	44
20.76				56051				22.56	45
20.78				56119	-25.9	295	68	22.35	45
20.80				56187				23.25	45
20.82				56255	-26.8	347	69	24.21	45
20.84				56323				23.64	44

20.86					56390	-26.8	347	69	22.82	45
20.88					56458				22.97	45
20.90					56526	-27.2	370	70	22.48	45
20.92					56594				22.64	44
20.94					56662	-25.5	272	68	24.32	44
20.96					56730				21.44	44
20.98					56797	-26.6	336	69	24.24	43
21.00					56865				24.65	43
21.02					56933	-26.6	336	69	23.86	42
21.04					57001				23.55	41
21.06					57069	-27.4	382	71	23.96	41
21.08					57136				24.95	40
21.10					57204	-26.5	330	69	24.30	40
21.12					57272				25.16	39
21.14					57340	-26.3	318	68	26.22	39
21.16					57408				24.98	39
21.18					57476	-26.0	301	68	25.72	38
21.20					57543				25.29	38
21.22					57611	-27.0	359	70	25.62	37
21.24					57679				25.25	37
21.26					57747	-26.8	347	69	25.31	36
21.28					57815				26.78	36
21.30					57882	-25.3	260	67	27.17	35
21.32					57950				25.52	35
21.34					58018	-25.3	260	67	25.59	34
21.36					58086				26.87	33
21.38					58154	-26.5	330	69	27.24	32
21.40					58221				29.09	32
21.42					58289	-26.8	347	69	27.56	31

21.44			58357				28.98	30
21.46			58425	-26.1	307	68	30.00	30
21.48			58493				30.19	30
21.50			58561	-25.1	248	67	29.32	30
21.52			58628				29.81	29
21.54			58696	-25.3	260	67	27.43	29
21.56			58764				30.85	29
21.58			58832	-26.5	330	69	28.88	29
21.60			58900				28.84	29
21.62			58967	-26.4	324	69	32.65	29
21.64			59035				29.29	29
21.66			59103	-25.5	272	68	31.46	29
21.68			59171				29.59	29
21.70			59239	-26.2	312	68	31.38	29
21.72			59307				33.27	29
21.74			59374	-26.8	347	69	30.85	29
21.76			59442				31.64	29
21.78	59510	H6	59510	-26.4	324	69	33.06	29
21.80			59554				31.68	29
21.82			59599	-24.7	225	68	31.97	29
21.84			59643				29.53	29
21.86			59688	-26.3	318	68	28.90	29
21.88			59732				30.59	29
21.90			59776	-25.2	254	67	32.64	28
21.92			59821				32.76	29
21.94			59865	-25.3	260	67	31.25	29
21.96			59910				29.65	29
21.98			59954	-25.3	260	67	30.02	29
22.00			59998				31.20	29

22.02					60043	-25.6	278	68	30.90	29
22.04					60087				29.01	28
22.06					60132	-24.9	237	67	31.42	28
22.08					60176				27.95	28
22.10					60220	-24.8	231	68	29.16	28
22.12					60265				30.95	28
22.14					60309	-25.8	289	68	30.21	28
22.16					60354				29.68	28
22.18					60398	-26.1	307	68	30.87	28
22.20					60442				32.16	28
22.22					60487	-25.7	283	68	31.43	28
22.24					60531				31.98	28
22.26					60576	-25.8	289	68	32.36	28
22.28					60620				29.84	28
22.30					60664	-25.1	248	67	29.80	28
22.32					60709				30.66	28
22.34					60753	-25.0	243	67	30.27	28
22.36					60798				30.87	28
22.38					60842	-25.2	254	67	30.91	28
22.40					60886				29.14	28
22.42					60931	-24.4	208	68	29.52	28
22.44					60975				30.69	28
22.46					61020	-24.5	214	68	30.98	28
22.48					61064				31.53	28
22.50					61108	-26.4	324	69	30.12	29
22.52					61153				30.73	28
22.54					61197	-25.4	266	67	30.51	28
22.56					61242				32.28	28
22.58					61286	-25.8	289	68	30.93	29

22.60	61330				30.24	29
22.62	61375	-24.7	225	68	31.92	29
22.64	61419				31.07	28
22.66	61464	-25.7	283	68	30.71	29
22.68	61508				31.36	29
22.70	61552	-25.0	243	67	29.98	29
22.72	61597				29.81	29
22.74	61641	-25.2	254	67	30.67	29
22.76	61686				30.98	29
22.78	61730	-26.3	318	68	29.50	29
22.80	61774				30.03	29
22.82	61819	-25.7	283	68	32.19	29
22.84	61863				31.70	28
22.86	61908	-25.2	254	67	29.07	28
22.88	61952				29.81	28
22.90	61996	-24.6	219	68	32.08	28
22.92	62041				30.02	28
22.94	62085	-25.9	295	68	29.46	28
22.96	62130				30.90	28
22.98	62174	-25.3	260	67	29.02	28
23.00	62218				30.36	28
23.02	62263	-25.7	283	68	30.67	28
23.04	62307				29.92	28
23.06	62352	-25.1	248	67	30.78	28
23.08	62396				30.12	28
23.10	62440	-24.5	214	68	30.13	28
23.12	62485				29.02	28
23.14	62529	-25.0	243	67	30.30	29
23.16	62574				29.49	28

23.18					62618	-25.8	289	68	29.60	29
23.20					62662				29.94	28
23.22					62707	-24.6	219	68	29.32	28
23.24					62751				29.46	28
23.26					62796	-25.8	289	68	28.81	28
23.28					62840				27.77	28
23.30					62884	-25.9	295	68	29.34	28
23.32					62929				29.23	28
23.34					62973	-25.2	254	67	30.59	28
23.36					63018				30.02	27
23.38					63062	-24.3	202	68	27.05	28
23.40					63106				29.49	28
23.42					63151	-25.6	278	68	28.77	28
23.44					63195				29.48	28
23.46					63240	-25.7	283	68	30.13	28
23.48					63284				29.51	28
23.50					63328	-26.8	347	69	29.01	28
23.52					63373				29.76	28
23.54					63417	-26.6	336	69	28.00	28
23.56					63462				29.05	28
23.58					63506	-25.5	272	68	30.41	28
23.60					63550				29.59	28
23.62					63595	-26.3	318	68	30.20	28
23.64					63639				29.87	28
23.66					63684	-26.2	312	68	29.22	28
23.68					63728				28.54	28
23.70					63772	-25.9	295	68	29.92	28
23.72					63817				28.93	28
23.74					63861	-25.5	272	68	29.44	28

23.76				63906				27.74	28
23.78		63950	IS18	63950	-26.4	324	69	25.08	28
23.80				64016				27.15	28
23.82				64082	-26.5	330	69	28.11	28
23.84				64148				27.56	27
23.86				64214	-25.0	243	67	27.40	27
23.88				64280				27.87	27
23.90				64346	-25.8	289	68	27.31	27
23.92				64412				27.06	28
23.94				64479	-26.5	330	69	27.13	28
23.96				64545				26.21	28
23.98				64611	-26.8	347	69	28.46	28
24.00				64677				26.10	28
24.02				64743	-24.0	185	68	26.60	28
24.04				64809				27.75	28
24.06				64875	-26.9	353	70	27.93	28
24.08				64941				25.91	27
24.10				65007	-26.6	336	69	26.72	27
24.12				65073				26.71	27
24.14				65139	-26.9	353	70	26.89	27
24.16				65205				27.94	27
24.18				65271	-26.1	307	68	27.49	27
24.20				65337				28.15	26
24.22				65404	-26.5	330	69	28.28	27
24.24				65470				28.45	27
24.26				65536	-25.3	260	67	28.30	28
24.28				65602				30.20	27
24.30				65668	-25.5	272	68	28.21	28
24.32				65734				30.35	28

24.34					65800	-24.7	225	68	30.00	28
24.36					65866				29.73	28
24.38					65932	-24.6	219	68	29.56	28
24.40					65998				29.76	28
24.42					66064	-25.8	289	68	29.67	28
24.44					66130				29.56	28
24.46					66196	-25.7	283	68	30.09	28
24.48					66262				28.87	28
24.50					66329	-26.1	307	68	29.46	28
24.52					66395				28.57	28
24.54					66461	-24.6	219	68	28.21	28
24.56					66527				28.75	28
24.58					66593	-25.5	272	68	29.66	28
24.60					66659				29.85	29
24.62					66725	-26.0	301	68	29.53	29
24.64					66791				30.49	28
24.66					66857	-24.6	219	68	29.00	28
24.68					66923				29.89	27
24.70					66989	-24.9	237	67	29.85	27
24.72					67055				29.73	27
24.74					67121	-24.4	208	68	29.13	27
24.76					67187				29.09	27
24.78					67254	-24.7	225	68	28.92	27
24.80					67320				29.35	27
24.82					67386	-25.4	266	67	29.19	27
24.84					67452				29.47	27
24.86					67518	-25.5	272	68	29.47	27
24.88					67584				28.68	27
24.90					67650	-26.0	301	68	29.80	27

24.92			67716				28.46	27
24.94			67782	-25.4	266	67	30.07	27
24.96			67848				29.38	27
24.98			67914	-26.0	301	68	30.44	27
25.00			67980				29.51	27
25.02			68046	-25.0	243	67	30.77	27
25.04			68112				30.20	27
25.06			68179	-25.8	289	68	30.75	27
25.08			68245				30.54	28
25.10			68311	-25.3	260	67	29.55	28
25.12			68377				29.59	27
25.14			68443	-26.7	341	69	28.07	28
25.16			68509				29.77	27
25.18			68575	-25.2	254	67	29.94	28
25.20			68641				29.36	28
25.22			68707	-26.6	336	69	29.90	27
25.24			68773				29.43	28
25.26			68839	-24.5	214	68	30.22	28
25.28			68905				28.79	28
25.30			68971	-25.7	283	68	27.71	28
25.32			69037				30.98	28
25.34			69104	-26.1	307	68	30.26	28
25.36			69170				28.73	27
25.38			69236	-26.0	301	68	26.27	27
25.40			69302				28.84	27
25.42			69368	-26.6	336	69	28.64	27
25.44			69434				29.60	27
25.46	69500	IS19	69500	-27.5	388	71	30.31	27
25.48			69566				28.81	27

25.50					69632	-27.3	376	71	28.66	27
25.52					69698				30.24	27
25.54					69764	-26.4	324	69	28.51	27
25.56					69830				29.70	27
25.58					69896	-25.8	289	68	28.21	28
25.60					69962				28.05	27
25.62					70029	-25.6	278	68	29.17	27
25.64					70095				29.86	27
25.66					70161	-25.1	248	67	26.86	27
25.68					70227				30.41	27
25.70					70293	-24.7	225	68	28.76	27
25.72					70359				27.55	27
25.74					70425	-25.4	266	67	29.39	27
25.76					70491				28.82	27

Part 8, climate data from Jingyuan and Linxia (1961~1990)

The climate data from Jingyuan and Linxia (close to the YB profile) stations, including the monthly precipitation and temperature data from 1961 t0 1990, are shown in Table S3 as following:

Table S3 climate data from Jingyuan and Linxia stations (1961~1990).

**Precipitation from Jingyuan station (Longitude E 104.67°, Latitude N 36.57°, Altitude 1397.8m a.s.l.)**													
**Years**	**Jan**	**Feb**	**Mar**	**Apr**	**May**	**Jun**	**Jul**	**Aug**	**Sep**	**Oct**	**Nov**	**Dec**	**MAP**
**1961**	0.3	1.9	8.3	20.1	16	19.3	35.4	63.9	63.9	42.9	13	0	285
**1962**	0.2	0	0	0.7	20	3.8	43.2	10.7	74.9	34.5	11.5	0	199.5
**1963**	0	4.4	5.3	13.7	30.2	26.2	53.1	7.9	33	12.3	1	0	187.1
**1964**	6.2	0.7	12.4	20.7	32.3	32	88.8	95.8	51.6	17.2	0.6	0.3	358.6
**1965**	0	0.2	0.8	9.4	35.8	31.3	23.7	25.2	19.9	42	5.3	0	193.6
**1966**	0.3	3	0	8.9	12.7	5.1	47.4	56.1	38.2	25.7	11.6	0	209
**1967**	0	0	17.5	41.6	85.2	30.6	66.2	75.2	24.9	16.2	14.1	0	371.5
**1968**	1.6	0.4	6.7	28	2.5	5	57.4	84.4	10.4	29.2	10.7	0	236.3
**1969**	0.6	1.5	3.6	0.6	28.6	13.1	19.8	53.1	52.8	14.1	4.4	0	192.2
**1970**	0	4.9	2.6	22.4	12.2	21.6	106.1	72.7	28.3	8.7	1	0	280.5
**1971**	0.8	3.1	3.3	4.2	35.3	34.5	25.4	75	49.3	17.2	9	0.6	257.7
**1972**	0.3	11.7	15.2	11.7	38.4	19.8	16.8	49.4	4.3	5	1.2	0	173.8
**1973**	0.9	0.1	4.2	5.5	50.5	25.7	41.1	71.4	41.3	13.3	0.1	0	254.1
**1974**	1.5	5.9	13.8	5.4	25.8	26.7	28.5	35.5	22.7	14.9	5.6	3.4	189.7
**1975**	0.7	0.2	0.9	12.5	29.4	13.4	41.9	41.8	14.4	33	5.6	3.5	197.3
**1976**	0	3.3	1.8	7.5	14	23.4	73.1	94.4	37.2	0	0	0	254.7
**1977**	1	2.3	1.2	31.4	13.3	21.3	85.7	34.2	28.3	19.8	10.1	0.1	248.7
**1978**	1.8	3.3	14.8	8	27.6	27.2	94.3	86.9	60.7	46.2	1.5	1.6	373.9
**1979**	1.1	0.5	2.4	8	0.7	26.4	105.7	63.1	35	4	0.7	0	247.6
**1980**	0.1	0.4	2.9	11.5	13.2	18.4	26.8	23.1	22.3	16.5	0	0.2	135.4
**1981**	1.2	0.5	0	24.3	0.2	28.2	43.7	28	24.6	4.4	0.2	0.2	155.5
**1982**	1.1	3.7	0.7	1.4	22.7	17.4	9.4	31	40.2	7.4	1.9	0	136.9
**1983**	1.2	0.1	2.7	26.4	29.5	17.4	8.7	64	50.9	22.7	0	0	223.6
**1984**	0.5	0	0	20.8	60.2	35.5	39.1	84.3	18.7	1.2	0	1.7	262
**1985**	0	0	3.3	6.3	63.3	53	49.8	111.1	78.4	51.6	0	0	416.8
**1986**	0	0	1.1	2.9	34.5	106.9	21.7	40.2	0.8	1.4	1.5	3.5	214.5
**1987**	0	2.1	6.1	16.2	33.3	25.2	25.6	35.9	34.2	5.3	1.5	0.1	185.5
**1988**	0	2.3	6.2	0	61.5	31.6	48.7	44.1	46.5	21.8	0	2.9	265.6
**1989**	2.4	6.1	1.1	41.1	1.1	27.7	11.2	56.2	20.2	28.5	7.8	0.3	203.7
**1990**	2.5	2.1	15.4	9.8	27.7	23.4	33	81.7	26	24.7	0	0	246.3
**Average**	0.9	2.2	5.1	14.0	28.6	26.4	45.7	56.5	35.1	19.4	4.0	0.6	238.6
**Temperature from Jingyuan station (Longitude E 104.67°, Latitude N 36.57°, Altitude 1397.8m a.s.l.)**													
**Years**	**Jan**	**Feb**	**Mar**	**Apr**	**May**	**Jun**	**Jul**	**Aug**	**Sep**	**Oct**	**Nov**	**Dec**	**MAT**

**1961**	-9.4	-6.3	0.3	7.9	11.7	15.7	17.8	16.1	11.1	6.2	-1.4	-6.8	5.2
**1962**	-9.3	-3.5	0	6.1	11.5	14.4	16.1	16.2	11.4	6.5	-2.5	-6.4	5
**1963**	-9.9	-4.6	2.4	7.4	12.6	14.1	16.1	16.8	12.5	6.4	-0.4	-6.5	5.6
**1964**	-9.9	-6.7	2.7	9.4	11.4	13.4	15.5	15.7	11.6	7.5	-0.5	-7	5.3
**1965**	-6.9	-4.6	0.8	7.9	11.2	13.7	15.8	14.7	11.6	5.8	-0.7	-8.7	5.1
**1966**	-7.2	-3.8	2.8	7.3	11.6	15.4	16.3	16.5	10	5.4	-2.5	-6.9	5.4
**1967**	-9.7	-5.9	1	5.4	12	13.6	16.5	15.6	10.5	5.6	-2.8	-10	4.3
**1968**	-7.7	-7.6	1.8	7	11.1	14.5	15.6	14.2	11.5	5.2	0	-4.8	5.1
**1969**	-8.1	-6.5	2.2	7.1	12.9	13.8	16.9	15.8	11.4	7.3	-2.5	-7.9	5.2
**1970**	-10.4	-5	-1.5	4.7	12.3	12.9	16.8	15.7	11.1	5.3	-0.9	-5.6	4.6
**1971**	-9.2	-4.5	2.9	7.7	10.5	14	17.8	16.4	10.6	5.5	-1.1	-5.6	5.4
**1972**	-6.5	-8.7	2.7	6.5	11.9	14	15.8	17	10.7	4.8	-1.9	-6.8	5
**1973**	-7.3	-2.5	2.4	8.3	10.4	13	15.4	15.9	11.5	5.3	-0.7	-7	5.4
**1974**	-8.2	-6.8	-0.6	7.8	11.3	14.4	16.3	15.6	10.9	5.1	0.5	-8.2	4.8
**1975**	-7.5	-4.8	2.5	7.2	10.1	13.9	15.5	16	12.3	6.5	-0.9	-10.6	5
**1976**	-8.4	-4	-1.2	6	11.2	13.8	14.6	13.7	11.3	5.9	-3	-7.9	4.3
**1977**	-10.2	-7.2	0.6	6.6	9.8	13.1	16.5	15.5	11.9	6.1	-2	-5.3	4.6
**1978**	-9.5	-5.5	0.6	7.9	12.4	14	16	15.8	10.6	5.9	0	-3.8	5.4
**1979**	-7.1	-3	0.4	7.6	11.1	14.6	15	15.4	11.3	6.6	-2.2	-5.3	5.4
**1980**	-8.6	-6.3	1.6	7.8	11.8	13.6	16	16	11.5	7	0.7	-6.2	5.4
**1981**	-8.1	-3.8	2.6	7.6	11.8	14.7	17.3	16.1	11.5	4.2	-1.6	-8.7	5.3
**1982**	-5.7	-3.8	1.6	5.3	11.1	14.1	16.4	16.2	10.9	7.4	-0.9	-8.7	5.3
**1983**	-10	-5.9	-0.5	5.4	12	13.8	15.4	15.8	12.3	6.8	0.3	-7.3	4.8
**1984**	-11	-5.9	0.6	7	11	14.3	15.3	15.2	11.1	5.2	0.6	-8	4.6
**1985**	-7.7	-3.6	0	8.3	12	13.5	15.3	15.9	10.5	6.2	-1.4	-6.8	5.2
**1986**	-8.3	-4.7	-0.3	6.3	12.1	13.5	15.5	15.2	11.4	6.2	-1.4	-4.3	5.1
**1987**	-5.8	-2	2.4	8.1	10.2	14.3	16.2	16	11.7	6.7	0	-5.3	6
**1988**	-6.6	-5.3	-1.2	7	11.4	14.5	17.4	16.1	11.6	6.1	0.1	-4.9	5.5
**1989**	-8.3	-5.8	-0.1	6.2	11.8	14.8	16.5	15.3	12.4	7.2	-0.3	-4.2	5.5
**1990**	-6.8	-5.1	2.8	5.9	10.8	14.4	16.7	16.4	12.8	6.4	1.2	-4.8	5.9
**Average**	-8.3	-5.1	1.1	7.0	11.4	14.1	16.1	15.8	11.4	6.1	-0.9	-6.7	5.2

**Precipitation from Linxia station (Longitude E 103.18°, Latitude N 35.62°, Altitude 1917m a.s.l.)**													
**Years**	**Jan**	**Feb**	**Mar**	**Apr**	**May**	**Jun**	**Jul**	**Aug**	**Sep**	**Oct**	**Nov**	**Dec**	**MAP**
**1961**	3.2	3.7	34.3	14.8	27	46.9	81	147.8	132.3	89	11.2	1.5	592.7
**1962**	0.5	1.9	2.7	21.2	20.3	47.4	81.9	50.5	74.8	69.5	15.3	0.7	386.7
**1963**	0	0.8	7.9	23.5	121.8	59.1	91.8	67.4	74.7	27	7	2.3	483.3
**1964**	6.6	4.2	19.3	62.2	87.9	83.6	134.3	177.6	116.7	39.1	0	0.7	732.2
**1965**	0	1.1	3.4	90	55.5	37.1	84.2	32	34.9	26.8	3.1	1.9	370
**1966**	0	1.5	5.9	25.6	54.1	33.2	51.8	79.5	102.5	38.9	3.2	0.4	396.6
**1967**	0.9	4.3	36.5	52.1	167.3	51.7	156	124.4	107.9	47.2	15.3	0	763.6
**1968**	1	1.9	11.1	27.1	45.4	32.3	83.2	126.3	68	42.4	4.9	0.2	443.8
**1969**	0	0.8	10	25.6	68.7	35	33.3	76.8	56.8	15.8	2.5	0.5	325.8
**1970**	4.8	5.1	12.6	62.3	38.8	71.3	89.6	217.1	86.3	21.8	0.6	0	610.3
**1971**	3.2	1.8	12.8	13.7	67.4	17.5	144.2	80.7	94.2	35.1	9.6	0	480.2
**1972**	0.4	9.9	6	45.2	54.5	64.8	70.2	93.2	8.3	7.8	8.6	0.7	369.6

**1973**	1.6	0	7.9	49.3	101.7	85.8	111.3	169.7	63.4	35.2	9.7	0	635.6
**1974**	1.9	9.4	18.7	29.9	48.8	23.6	82.5	42	82	57.3	11.5	2.1	409.7
**1975**	0.3	9	6.9	11.9	68	35.2	107.8	116.2	120	76.6	10.8	2.8	565.5
**1976**	0	6.4	6.3	13.1	70.7	101.2	65.3	209.4	99.3	13.8	0.6	0	586.1
**1977**	2.8	3.9	3.2	56.8	62.2	23.3	87.8	92	33.7	54.4	30	3.3	453.4
**1978**	2.8	0.7	27.4	17.8	36.4	78.2	135.5	84.3	115.4	85.4	9.4	0.9	594.2
**1979**	3.3	3.2	12	13.7	22.3	55.2	142.9	177.4	80.7	12.8	15.7	2.6	541.8
**1980**	5.7	5.8	8.3	18.1	27.1	40.7	91	101.8	61.4	5	0	0	364.9
**1981**	6.4	0.7	16.6	57	4.8	77.9	161.3	162.8	80.2	11.4	9.9	2.7	591.7
**1982**	1.4	3.4	35.9	50.8	38.7	33.4	26.8	82.5	106.6	36.6	11.2	1.5	428.8
**1983**	11.4	2.8	13	60.8	25.3	55.7	95.9	108.5	64.5	53.6	0	1.9	493.4
**1984**	3.6	2.4	7.2	39.9	82.2	109.8	78.3	100.8	121.1	36.1	0.3	3.6	585.3
**1985**	1.7	0.1	6.7	22.4	108.2	93.4	68.8	66.4	75.5	55.9	0.5	3.4	503
**1986**	0.7	2.8	20.5	15.3	82.1	117.9	50.8	96.6	15.9	16.8	4.2	2.9	426.5
**1987**	0.2	3.4	7.4	37	97.7	123.4	49.5	22.3	54.7	15.5	0.5	0.3	411.9
**1988**	0.5	12.2	28.4	18.9	90.3	76.6	72.3	85.8	86.1	39.5	0.5	0.6	511.7
**1989**	3.7	14.6	16.7	60.8	9.6	59.5	139.8	74.1	51.2	54.5	4	5.5	494
**1990**	3.3	10	24.8	67.5	76.2	40.3	106.6	77.6	55.4	46.1	2.2	0	510
**Average**	2.4	4.3	14.3	36.8	62.0	60.4	92.5	104.8	77.5	38.9	6.7	1.4	502.1

Temperature from Linxia station (Longitude E 103.18°, Latitude N 35.62°, Altitude 1917m a.s.l.)

Years	Jan	Feb	Mar	Apr	May	Jun	Jul	Aug	Sep	Oct	Nov	Dec	MAT
**1961**	-8.4	-4.9	1.8	9.6	13.4	17.3	19.5	17.7	12.7	7.5	0.6	-5.6	6.8
**1962**	-8	-2.4	1.3	7.4	13.3	16.1	17.7	18.6	13.1	7.9	-1.1	-4.6	6.6
**1963**	-8.5	-3.7	4.2	8.7	14.2	15.5	17.7	18.8	14.1	7.9	1	-5.2	7.1
**1964**	-8.6	-5.8	4.4	11.1	13	15	17	17.9	13.2	9.2	1	-5.1	6.9
**1965**	-5.7	-3	2.8	9.8	13.1	15.8	17.6	17.1	13.8	8	1.2	-7	7
**1966**	-6	-2.2	4.3	9.3	13.4	17.2	18	18.4	11.6	7	-1.3	-6.1	7
**1967**	-8	-4.6	2.3	7.4	13.7	15.4	18.4	17.2	12.4	7.2	-1.5	-7.8	6
**1968**	-6.8	-6	3.1	8.7	12.9	16.7	17.4	16	13.3	6.5	1.5	-3.6	6.6
**1969**	-6.7	-5.1	3.6	9.5	15.1	15.9	18.8	17.4	13.3	9.1	-0.6	-6.3	7
**1970**	-8	-3.2	0.3	6.5	14.1	14.8	18.8	17.4	12.7	6.9	0.7	-4.4	6.4
**1971**	-7.2	-2.8	4.2	9.6	12.6	15.9	19.8	18.2	12.2	6.9	0.7	-4.3	7.2
**1972**	-5.2	-6.9	4	8.3	13.9	16.1	18.1	19.3	12.9	6.6	0.6	-5.4	6.9
**1973**	-5.8	-1.2	4	10.1	12.2	14.9	17.3	17.9	13.4	6.8	1.3	-5.3	7.1
**1974**	-6.5	-5.4	0.9	9.8	13.4	16.6	18.2	17.7	12.4	6.3	1.9	-6.4	6.6
**1975**	-5.9	-3	3.7	8.9	11.9	15.9	17.4	18.1	13.8	7.9	0.5	-8.7	6.7
**1976**	-6.8	-2.4	0.7	8.2	13.2	15.7	16.5	15.3	12.8	7.7	-1.3	-6.7	6.1
**1977**	-8.9	-5	2.5	8.6	12	15.1	18.4	17.5	13.8	7.9	0.2	-3.8	6.5
**1978**	-8	-4.2	2.1	9.9	14.6	16.1	17.9	17.5	12.3	7.4	1.7	-2.4	7.1
**1979**	-5.6	-1.6	2.1	9.3	13.2	16.7	16.6	16.8	12.7	8.2	-0.7	-4.1	7
**1980**	-7.3	-4.7	2.9	9.4	13.6	15.4	18	17.3	13.1	8.5	2.4	-4.7	7
**1981**	-6.9	-2.8	4.4	9.2	13.3	16.3	19.1	17.6	12.8	5.5	-0.2	-6.7	6.8
**1982**	-4.9	-2.5	3.2	7.3	12.7	15.5	18.2	18.1	12.3	8.7	0.6	-7.3	6.8
**1983**	-8.2	-4.4	0.9	7.1	13.6	15.5	16.9	17.4	13.8	8.1	1.5	-5.6	6.4
**1984**	-8.6	-4.5	1.8	8.8	12.6	15.7	17.1	17.1	12.5	6.8	1.9	-6.7	6.2

**1985**	-6.7	-2.5	1.3	10.2	13.7	15	16.9	18	12.1	7.4	0.3	-5.5	6.7
**1986**	-6.6	-3.4	1.1	8.3	14.1	15.1	17.4	17.2	13.2	7.5	-0.2	-3.3	6.7
**1987**	-5	-0.8	3.6	9.8	11.6	15.6	18.3	18.1	13.5	7.7	1.3	-4.1	7.5
**1988**	-5.9	-4	0.3	8.6	13	16.1	18.9	17.8	13.1	7.3	1.3	-3.6	6.9
**1989**	-6.9	-4.4	1.7	8.1	13.7	16.5	18.1	16.9	13.8	8.2	1.1	-2.8	7
**1990**	-5.5	-3.5	4.5	7.5	12.5	16.3	18.7	17.8	14	7.6	2.4	-3.8	7.4
**Average**	-6.9	-3.7	2.6	8.8	13.3	15.9	18.0	17.6	13.0	7.5	0.6	-5.2	6.8

Part 9, supplementary references

Amante, C. & Eakins, B. W. ETOPO1 1 Arc-Minute Global Relief Model: Procedures, Data Sources and Analysis. NOAA Technical Memorandum NESDIS NGDC-24, 19 (2009).

Chen, F. H., Bloemendal, J., Wang, J. M., Li, J. J. & Oldfield, F. High-resolution multi-proxy climate records from Chinese Loess: Evidence for rapid climatic changes over the last 75 kyr. *Palaeogeogr. Palaeoclimatol. Palaeoecol.* **130**, 323–335 (1997).

Chen, F. H., Rao, Z. G., Zhang, J. W., Jin, M. & Ma, J. Y. Variations of organic carbon isotopic composition and its environmental significance during the Last Glacial on western Chinese Loess Plateau. *Chin. Sci. Bull.* **51**, 1593–1602 (2006).

Chen, F. D. *et al*. Holocene moisture evolution in arid central Asia and its out-of-phase relationship with Asian monsoon history. *Quatern. Sci. Rev.* **27**, 351–364 (2008).

Diefendorf, A. F., Mueller, K. E., Wing, S. L., Koch, P. L. & Freeman, K. H. Global patterns in leaf ^{13}C discrimination and implications for studies of past and future climate. *Proc. Natl. Acad. Sci. U. S. A.* **107**, 5738–5743 (2010).

Dong, J. G. *et al*. A high-resolution stalagmite record of the Holocene East Asian monsoon from Mt Shennongjia, central China. *Holocene* **20**, 257–264 (2010).

Feng, Z. D. *et al*. Climatic dependency of soil organic carbon isotopic composition along the S–N Transect from 34°N to 52°N in central-east Asia. *Palaeogeogr. Palaeoclimatol. Palaeoecol.* **257**, 335–343 (2008).

Gu, Z. Y. *et al*. Climate as the dominant control on C_3 and C_4 plant abundance in the Loess

Plateau: Organic carbon isotope evidence from the last glacial-interglacial loess-soil sequences. *Chin. Sci. Bull.* **48**, 1271–1276 (2003).

Kohn, M. J. Carbon isotope compositions of terrestrial C3 plants as indicators of (paleo)ecology and (paleo)climate. *Proc. Natl. Acad. Sci. U. S. A.* **107**, 19691–19695 (2010).

Lai, Z. P. & Wintle, A. G. Locating the boundary between the Pleistocene and the Holocene in Chinese loess using luminescence. *Holocene* **16**, 893–899 (2006).

Lai, Z. P., Wintle, A. G. & Thomas, D. S. G. Rates of dust deposition between 50 ka and 20 ka revealed by OSL dating at Yuanbao on the Chinese Loess Plateau. *Palaeogeogr. Palaeoclimatol. Palaeoecol.* **248**, 431–439 (2007).

Lee, X. Q. *et al.* Carbon isotope of bulk organic matter: A proxy for precipitation in the arid and semiarid central East Asia. *Glob. Biogeochem. Cycle* **19**, GB4010 (2005).

Li, J. Z. *et al.* Variations in carbon isotope ratios of C_3 plants and distribution of C_4 plants along an altitudinal transect on the eastern slope of Mount Gongga. *Science In China Ser D-Earth Sci.* **52**, 1714-1723 (2009).

Liu, W. G. *et al.* $\delta^{13}C$ variation of C_3 and C_4 plants across an Asian monsoon rainfall gradient in arid northwestern China. *Glob. Change Biol.* **11**, 1094–1100 (2005a).

Liu, W. G. *et al.* Summer monsoon intensity controls C_4/C_3 plants abundance during the last 35 ka in the Chinese Loess Plateau: Carbon isotope evidence from bulk organic matter and individual leaf waxes. *Palaeogeogr. Palaeoclimatol. Palaeoecol.* **220**, 243–254 (2005b).

Liu, W. G. *et al.* Carbon isotopic composition of modern soil and paleosol as a response to vegetation change on the Chinese Loess Plateau. *Sci. China Ser. D-Earth Sci.* **48**, 93–99 (2005c).

Liu, W. G., Yang, H., Sun, Y. B. & Wang, X. L. $\delta^{13}C$ values of loess total carbonate: A

sensitive proxy for Asian summer monsoon in arid northwestern margin of the Chinese loess plateau. *Chem. Geol.* **284**, 317–322 (2011).

Long, S. P. C_4 photosynthesis at low temperatures. *Plant. Cell. Environ.* **6**, 345-363 (1983).

North Greenland Ice Core Project (NGRIP) members, High-resolution record of Northern Hemisphere climate extending into the last interglacial period. *Nature* **431**, 147–151 (2004).

Porter, S. C. & An, Z. S. Correlation between climate events in the North-Atlantic and China during last glaciation. *Nature* **375**, 305–308 (1995).

R Core Team. R: A language and environment for statistical computing. R Foundation for Statistical Computing, Vienna, Austria. (http://www.R-project.org/, 2013).

Rao, Z. G., Chen, F. H., Cao, J., Zhang, P. Z. & Zhang, P. Y. Variation of soil organic carbon isotope and C_3/C_4 vegetation type transition in the western Loess Plateau during the last glacial and Holocene periods. *Quatern. Sci.* **25**, 107−114 (2005) (in Chinese with English abstract).

Rao, Z. G., Zhu, Z. Y., Chen, F. H., Zhang, J. W. Does $\delta^{13}C_{carb}$ of the Chinese loess indicate past C_3/C_4 abundance? A review of research on stable carbon isotopes of the Chinese loess. *Quatern. Sci. Rev.* **25**, 2251–2257 (2006).

Rao, Z.G. *et al*. Relationship between climatic conditions and the relative abundance of modern C_3 and C_4 plants in three regions around the North Pacific. *Chin. Sci. Bull.* **55**, 1931–1936 (2010).

Rao, Z.G. *et al*. Spatial and temporal variations of C_3/C_4 relative abundance in global terrestrial ecosystem since the Last Glacial and its possible driving mechanisms. *Chin. Sci. Bull.* **57**, 4024–4035 (2012).

Stewart, G. R., Turnbull, M. H., Schmidt, S., and Reskine, P. D. ^{13}C natural abundance in plant communities along a rainfall gradient: A biological integrator of water availability.

Aust. Journ. Plant Physi. **22**, 51–55 (1995).

Sun, Y. B., Wang, X. L., Liu, Q. S. & Clemens, S. C. Impacts of post-depositional processes on rapid monsoon signals recorded by the last glacial loess deposits of northern China. *Earth Planet. Sci. Lett.* **289**, 171–179 (2010).

Sun, Y. B. *et al.* Influence of Atlantic meridional overturning circulation on the East Asian winter monsoon. *Nat. Geosci.* **5**, 46–49 (2012).

Svensson, A., *et al*. A 60 000 year Greenland stratigraphic ice core chronology. *Clim. Past.* **4**, 47–57 (2008).

Vidic, N. J. & Montañez, I. P. Climatically driven glacial-interglacial variations in C_3 and C_4 plant proportions on the Chinese Loess Plateau. *Geology* **32**, 337–340 (2004).

Wang, G. A. & Han, J. M. Relations between $\delta^{13}C$ values of C_3 plants in northwestern China and annual precipitation. *Chin. Journ. Geol.* **36**, 494-499 (2001a) (in Chinese with English abstract).

Wang, G. A. & Han, J. M. $\delta^{13}C$ variations of C_3 plants in dry and rainy seasons. *Marine Geol. & Quatern. Geol.* **21**, 43-47 (2001b) (in Chinese with English abstract).

Wang, G. A., Han, J. M. & Liu, T. S. The carbon isotope composition of C_3 herbaceous plants in loess area of northern China. *Sci. China Ser. D-Earth Sci.* **46**, 1069–1076 (2003).

Wang, G. A. *et al*. Altitudinal trends of leaf $\delta^{13}C$ follow different patterns across a mountainous terrain in north China characterized by a temperate semi-humid climate. *Rapid Commu. Mass Spectro.* **24**, 1557-1564 (2010).

Zhang, Z. H., Zhao, M. X., Lu, H. Y., & Faiia, A. M. Lower temperature as the main cause of C_4 plant declines during the glacial periods on the Chinese Loess Plateau. *Earth Planet. Sci. Lett.* **214**, 467–481 (2003).

Zheng, S. X. & Shangguan, Z. P. Spatial patterns of foliar stable carbon isotope compositions of C_3 plant species in the Loess Plateau of China. *Ecol. Res.* **22**, 342–353 (2007).
